# Genomic landscape of the individual host response and outcomes in sepsis: a prospective cohort study

**DOI:** 10.1016/S2213-2600(16)00046-1

**Published:** 2016-04

**Authors:** Emma E Davenport, Katie L Burnham, Jayachandran Radhakrishnan, Peter Humburg, Paula Hutton, Tara C Mills, Anna Rautanen, Anthony C Gordon, Christopher Garrard, Adrian V S Hill, Charles J Hinds, Julian C Knight

**Affiliations:** a Wellcome Trust Centre for Human Genetics, University of Oxford, Oxford, UK; b Adult Intensive Care Unit, John Radcliffe Hospital, Oxford, UK; c Section of Anaesthetics, Pain Medicine and Intensive Care, Imperial College, London, UK; d William Harvey Research Institute, Barts and The London School of Medicine, Queen Mary University, London, UK

## Abstract

**Background:**

Effective targeted therapy for sepsis requires an understanding of the heterogeneity in the individual host response to infection. We investigated this heterogeneity by defining interindividual variation in the transcriptome of patients with sepsis and related this to outcome and genetic diversity.

**Methods:**

We assayed peripheral blood leucocyte global gene expression for a prospective discovery cohort of 265 adult patients admitted to UK intensive care units with sepsis due to community-acquired pneumonia and evidence of organ dysfunction. We then validated our findings in a replication cohort consisting of a further 106 patients. We mapped genomic determinants of variation in gene transcription between patients as expression quantitative trait loci (eQTL).

**Findings:**

We discovered that following admission to intensive care, transcriptomic analysis of peripheral blood leucocytes defines two distinct sepsis response signatures (SRS1 and SRS2). The presence of SRS1 (detected in 108 [41%] patients in discovery cohort) identifies individuals with an immunosuppressed phenotype that included features of endotoxin tolerance, T-cell exhaustion, and downregulation of human leucocyte antigen (HLA) class II. SRS1 was associated with higher 14 day mortality than was SRS2 (discovery cohort hazard ratio (HR) 2·4, 95% CI 1·3–4·5, p=0·005; validation cohort HR 2·8, 95% CI 1·5–5·1, p=0·0007). We found that a predictive set of seven genes enabled the classification of patients as SRS1 or SRS2. We identified cis-acting and trans-acting eQTL for key immune and metabolic response genes and sepsis response networks. Sepsis eQTL were enriched in endotoxin-induced epigenetic marks and modulated the individual host response to sepsis, including effects specific to SRS group. We identified regulatory genetic variants involving key mediators of gene networks implicated in the hypoxic response and the switch to glycolysis that occurs in sepsis, including HIF1α and mTOR, and mediators of endotoxin tolerance, T-cell activation, and viral defence.

**Interpretation:**

Our integrated genomics approach advances understanding of heterogeneity in sepsis by defining subgroups of patients with different immune response states and prognoses, as well as revealing the role of underlying genetic variation. Our findings provide new insights into the pathogenesis of sepsis and create opportunities for a precision medicine approach to enable targeted therapeutic intervention to improve sepsis outcomes.

**Funding:**

European Commission, Medical Research Council (UK), and the Wellcome Trust.

## Introduction

A clinical diagnosis of sepsis identifies a heterogeneous population of patients with a dysregulated systemic inflammatory host response to infection in the presence of organ dysfunction.^1^ Substantial individual variation in this response restricts therapeutic options and is a major contributing factor in the failure of clinical trials of immunomodulatory treatments for sepsis.^2^, ^3^ The dynamic nature of the response and the importance of both hyperinflammatory and immunosuppressed states is now recognised, but effective biomarkers to enable targeted therapy appropriate to the immune response state of the individual at the time of intervention remain elusive.^4^

The existence of genetic associations with susceptibility to and outcomes of infectious diseases, including sepsis, suggests a role for host genomic variation in the heterogeneity seen among patients with sepsis.^5^, ^6^ However the challenges of defining disease phenotypes and establishing the functional significance of any genetic associations have so far limited such studies. Most associations involve DNA sequence variants in non-coding DNA for which regulatory effects on gene expression have been proposed. To define genes modulated by genetic variation and to identify specific functional regulatory polymorphisms, associations between genetic variants (usually single-nucleotide polymorphisms [SNPs]) and differences in gene expression between individuals can be mapped as expression quantitative trait loci (eQTL).^7^ In leucocytes from healthy volunteers, we have shown that genomic variants modulate differences in gene expression between individuals, including the response to innate immune stimuli such as lipopolysaccharides,^8^ but their functional significance in most disease states, including sepsis, is unknown. In sepsis, an acute severe illness associated with profound alterations in physiological, metabolic, and immune function, the mapping of eQTL would enable interrogation of associations involving the host immune response and diverse signalling pathways.

**Research in context**

**Evidence before this study**

We searched PubMed for articles published before Sept 1, 2015, using the terms “sepsis” OR “severe sepsis” OR “septic shock” AND “gene expression profiling” OR “transcriptomic” OR “microarray analysis”. We did a second search with the terms “sepsis” AND “eQTL” OR “expression quantitative trait” OR “regulatory variant”. We only considered peer-reviewed, English language reports. We identified 34 studies done in humans that examined the sepsis response in circulating leucocytes and peripheral blood. Of these studies, 12 focused on paediatric or neonatal sepsis. A further 19 involved small sample sizes (<100 patients), and the remainder combined data from previously published cohorts. Additionally, most recruited a heterogeneous patient cohort in terms of cause of sepsis. Several studies have aimed to develop diagnostic or prognostic predictive models, as well as for the subclassification of patients, using gene expression in paediatric sepsis. Reviews summarise the relatively low level of concordance across transcriptomic profiling studies of sepsis and suggest that these discrepancies are partly due to the use of underpowered, heterogeneous cohorts. The identification of regulatory variants through eQTL mapping has previously focused on cells and tissues from healthy individuals including whole blood, different immune cell types, and immune-relevant conditions of cellular activation. We identified no published studies that used eQTL mapping in the context of a severe, acute illness such as sepsis.

**Added value of this study**

We present data for large discovery and validation cohorts of patients with sepsis resulting from a single, well-defined cause. We identified two distinct patient clusters within these cohorts based on global gene expression soon after admission to intensive care, one of which shows an immunosuppressed phenotype and is associated with higher early mortality. We developed a classification model based on the expression of a predictive gene set, which we validated in a prospectively recruited cohort. Cluster membership could not be accurately predicted on the basis of clinical covariates. We also show that underlying genetic variation affects the individual immune response transcriptomic signature in the context of disease. Our integrated, translational genomics approach enhances phenotypic resolution, and reveals opportunities for precision medicine.

**Implications of all the available evidence**

Our study and others show the substantial degree of heterogeneity in the sepsis response, demonstrating the need for improved patient stratification and a precision medicine-based approach to management. The differences we found between the sepsis response groups have important therapeutic implications, including for the development of novel interventions, identification of high risk patients, and individually targeted therapy, and for the conduct of clinical trials.

In this study, we investigate the genomic landscape of sepsis due to community-acquired pneumonia, the most common cause of sepsis, in adult patients after admission to the intensive care unit (ICU). We find evidence of transcriptional signatures soon after ICU admission that provide information about the immune response state of an individual patient and their prognosis. We further investigate interindividual heterogeneity in the transcriptomic response to sepsis by use of an eQTL mapping approach and identify context-specific regulatory genetic variants involving gene networks central to the pathogenesis of sepsis.

## Methods

### Participants

We recruited patients to the discovery and validation cohorts as part of the UK Genomic Advances in Sepsis (GAinS) study (NCT00131196) from 29 participating ICUs (appendix 1 p 20) between Feb 1, 2006, and Feb 20, 2014. Ethics approval was granted by national ethics committees and locally approved for individual participating centres. After obtaining informed consent from the patient or their legal representative, we enrolled adult patients (aged >18 years) who were admitted to the ICU with sepsis due to community-acquired pneumonia (appendix 1 p 2). We analysed gene expression for 270 patients in the discovery cohort and 114 patients in the validation cohort. The independent validation cohort consisted of patients with sepsis due to community-acquired pneumonia who were prospectively recruited to GAinS with the same inclusion and exclusion criteria as the discovery cohort. Equal numbers of survivors and non-survivors were selected for gene expression profiling in the validation cohort to maximise informativeness for outcome. The Acute Physiology and Chronic Health Evaluation II (APACHE II) scores on admission and the Sequential Organ Failure Assessment (SOFA) scores on days 1, 3, and 5 were calculated from data entered into the electronic case report form (eCRF) by the local investigators.^9^, ^10^

### Procedures

We obtained samples for gene expression profiling by rapidly isolating the total blood leucocyte population from whole blood samples (about 10 mL) taken following admission to ICU by use of the LeukoLOCK (Thermo Fisher Scientific, Waltham, MA, USA) depletion filter technology (appendix 1 p 2). We purified total RNA and used Illumina Human-HT-12 version 4 Expression BeadChips with 47 231 probes (Illumina, San Diego, CA, USA) to do genome-wide transcription profiling for the first available sample taken following ICU admission for each of 270 patients in the discovery cohort and 114 patients in the validation cohort (appendix 1 p 2). We purified genomic DNA from whole blood samples (appendix 1 p 3) and genotyped individuals in the discovery cohort with Illumina HumanOmniExpress BeadChips (730 525 SNPs; Illumina, San Diego, CA, USA).

### Bioinformatics and statistical analysis

After background subtraction, we used the variance stabilisation and normalisation method to transform and normalise the microarray data (appendix 1 p 2). We removed five samples from the discovery cohort and eight samples from the validation cohort because they failed quality control (appendix 1 p 2). We did differential gene expression analysis for 26 185 probes using limma (R package; appendix 1 p 3). We investigated for enrichment of biological pathways and functions, and for upstream regulators, using Ingenuity Pathway Analysis (IPA; Qiagen, Redwood City, CA, USA).

After quality control, we investigated interindividual heterogeneity in the sepsis leucocyte transcriptome in the discovery cohort of 265 patients by doing unsupervised hierarchical cluster analysis for the most variable 10% of probes. We defined groupings of transcriptional sepsis response signatures (SRS) by agglomerative hierarchical clustering based on Ward's method and Euclidean distance to initiate the appropriate number of groups, followed by consolidation of group membership using *k*-means (FactoMineR [R package], appendix 1 p 3). Increasing the number of clusters between two and four showed no reduction in the within-group sum of squares after application of the *k*-means procedure, which suggests that this range included the optimum number of defined clusters in the dataset.

We identified clinical covariates or genes for use in prediction models by fitting a sparse response model suitable for high dimensional data (GeneRave [R package], appendix 1 p 3).^11^ We fitted generalised linear models with group membership or mortality as the response variable and either gene expression or clinical covariates as predictors. We established misclassification rates for models by use of leave-one-out cross-validation.^12^

We analysed two human endotoxin tolerance datasets^13^, ^14^ (appendix 1 p 3) to define an endotoxin tolerance gene signature consisting of 398 genes (false discovery rate [FDR] <0·05, fold change [FC] >1·5), of which 331 were also measured in the patients with sepsis. We assessed enrichment of this signature in the genes differentially expressed between SRS groups using ROAST,^15^ a rotation-based gene set test that takes account of directionality in addition to significance.

To prospectively validate our findings, we first identified a predictive gene set to define SRS group membership in the discovery cohort by fitting a sparse response model to the gene expression data.^11^ We then analysed the independent validation cohort by selecting all non-survivors and an equal number of age-matched and sex-matched survivors for gene expression profiling. After quality control for gene expression, we included 54 survivors and 52 non-survivors in the analysis. We assigned group membership for individuals on the basis of the expression of the predictive gene set (appendix 1 p 2). Group membership and association with outcome was robust to the exclusion of 17 individuals with a long length of stay (>15 days).

We did quality control of the genotyping data at the sample and SNP level by use of PLINK version 1.07 (appendix 1 p 3). We mapped eQTL using an additive linear model to establish associations between SNPs and gene expression as assayed by specific probes (Matrix eQTL [R package], appendix 1 p 3). After quality control and processing (appendix 1 p 3), we tested 644 390 SNPs for association with 17 347 uniquely mapping probes in 240 patients in the discovery cohort. eQTL were defined as cis when the distance between the chromosomal position of the SNP and probe start site was less than 1 Mb. eQTL with distances greater than 2·5 Mb were defined as trans and we restricted the analysis of trans-eQTL to autosomes. We did principal component analysis on the gene expression matrix and included the first 30 principal components of the expression data as covariates in the eQTL analysis to take account of SRS group and limit other potential confounding factors. For the SRS group-specific eQTL, we included the first 25 principal components. We found that incorporation of principal components enhanced discovery of cis-eQTL, consistent with previous studies (appendix 1 p 3 and p 5).^8^, ^16^, ^17^ This approach identified more eQTL than did inclusion of specific clinical covariates such as cell count. We defined SRS-specific cis-eQTL based on FDR less than 0·01 in one SRS group and greater than 0·05 in the other. We compared sepsis-associated eQTL with an eQTL dataset from a meta-analysis of population controls generated from whole blood samples (appendix 1 p 3).^17^

We annotated expression-associated SNPs with chromatin features for lipopolysaccharide-stimulated monocytes previously identified by the Blueprint Consortium,^18^ including histone marks (H3K27ac, H3K4me1, and H3K4me3) and DNase I hypersensitivity (appendix 1 p 3). We compared the proportion of expression-associated SNPs overlapping each mark with the proportion for SNPs within 1 Mb of a probe (Fisher's exact test) and distance to the nearest mark (Mann-Whitney test).

### Role of the funding source

The funders of the study had no role in study design, data collection, data analysis, data interpretation, or writing of the report. The corresponding author had full access to all the data and the final responsibility to submit for publication.

## Results

**Figure 1:**
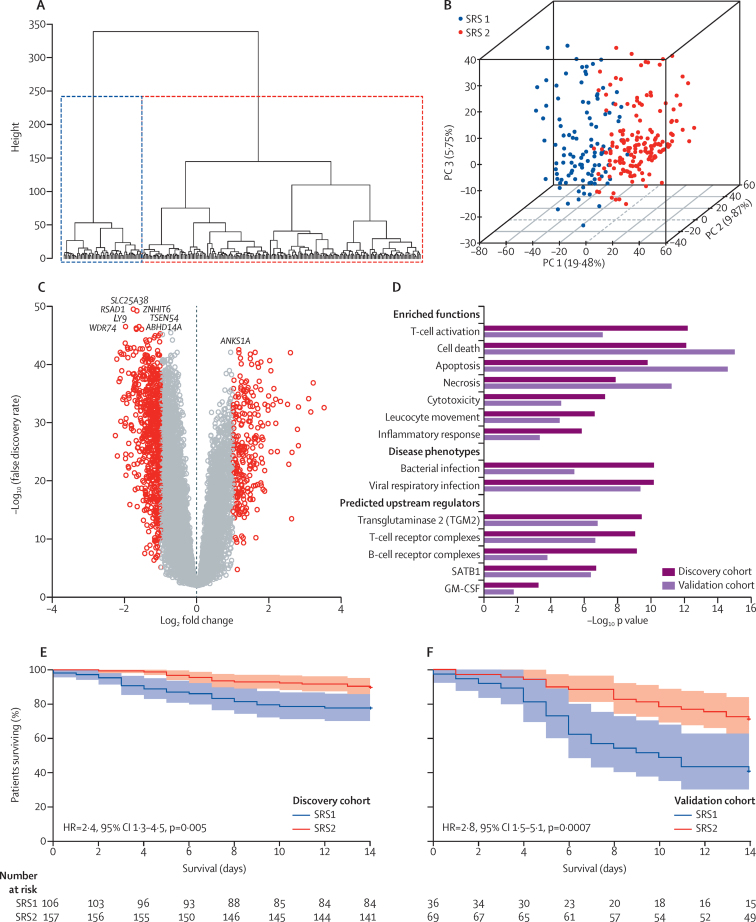
Transcriptomic sepsis response signatures Unsupervised hierarchical cluster analysis for the top 10% most variable probes (n=2619) for the 265 patients in the discovery cohort (A). First three PCs plotted with the proportion of variance explained by each component (B); individuals are coloured by group membership based on two groups assigned with *k*-means. Volcano plot (C) of differentially expressed probes for SRS1 versus SRS2 (red colouring shows fold change >1·5, false discovery rate <0·05). Most enriched functions, disease phenotypes, and predicted upstream regulators were derived from differentially expressed genes (D). Kaplan-Meier survival plot by SRS group (95% CIs shaded) for (E) discovery cohort and (F) validation cohort. SRS=sepsis response group. PC=principal component.

We first investigated interpatient heterogeneity in the transcriptomic response to sepsis for 265 patients in the discovery cohort; all patients showed evidence of organ dysfunction based on SOFA scores during ICU admission and 28 day mortality was 21% (table 1, appendix 2). Unsupervised hierarchical cluster analysis of global gene expression for peripheral blood leucocytes revealed two distinct clusters (figure 1A,B), which we denoted as sepsis response signature groups SRS1 and SRS2. 108 (41%) patients were assigned SRS1 and 157 (59%) patients as SRS2. We identified 3080 genes that were differentially expressed between groups (FC >1·5, FDR <0·05), with 2260 (73·4%) downregulated in SRS1 (figure 1C; appendix 2). We saw no differences between SRS groups in the expression of the pro-inflammatory cytokine genes *TNF, IL6*, or *IL1B*. However, pathway analysis of the differentially expressed genes showed functional differences related to T-cell activation, cell death, apoptosis, necrosis, cytotoxicity, and phagocyte movement. Specific predicted upstream regulators of the differentially expressed genes included transglutaminase II, B-cell and T-cell receptor complexes, SATB1, and GM-CSF (figure 1D, appendix 1 p 6, appendix 2).

We noted that SRS group membership was associated with outcome; specifically, SRS1 was significantly associated with higher early mortality (14 day mortality 22% for SRS1 *vs* 10% for SRS2, hazard ratio (HR) 2·4, 95% CI 1·3–4·5, p=0·005; figure 1E) and indicators of more severe illness, but not age, sex, or microbiology (table 2, appendix 2). In view of the differences that existed in white cell counts between patients with SRS1 and SRS2 (table 2), we also carried out differential gene expression analysis taking account of this (appendix 2). Pathway analysis showed that the enriched functions that we identified between SRS groups remained. We next investigated the extent to which clinical features might be predictive of SRS group (appendix 2). Any clinical covariate alone or in combination showed limited efficacy in prediction models for SRS group membership (misclassification rates using the most informative selected set of clinical covariates or SOFA or APACHE II scores, were 19·6%, 34·0%, and 41·1%, respectively, appendix 2). Similarly, the timing of sampling in relation to ICU admission was not predictive of SRS group membership (misclassification rate 40%).

Key mediators of endotoxin tolerance were differentially expressed between SRS groups, including increased expression of negative regulators of TLR signalling such as *IRAK3* and *TOLLIP* in patients with SRS1 (appendix 2). To further investigate the possible association with endotoxin tolerance, we used published data from endotoxin-naive and endotoxin-exposed (primed) peripheral blood mononuclear cells to define an endotoxin tolerance gene expression signature of 331 genes (FDR <0·05, FC >1·5).^13^, ^14^ We detected significant enrichment (p<1 × 10^−5^) of this endotoxin tolerance signature in genes differentially expressed in SRS1 relative to SRS2 (appendix 2).

**Figure 2:**
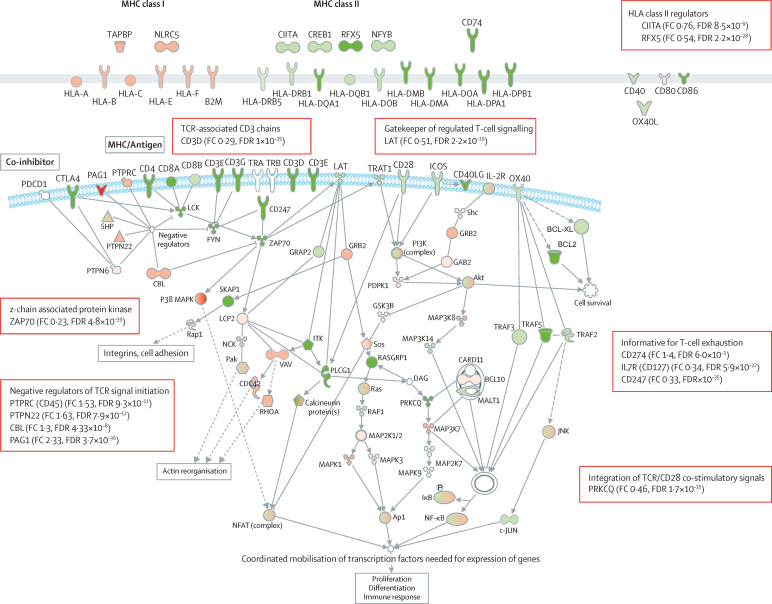
Overview of differentially expressed genes between SRS groups involving HLA and T-cell activation Red shading shows upregulation and green shading shows downregulation of genes (FC>1·5, FDR<0·05) for SRS1 versus SRS2. Solid lines show direct association, dashed lines show indirect association. SRS=sepsis response signature. FC=fold change. FDR=false discovery rate. MHC=major histocompatibility complex.

**Figure 3:**
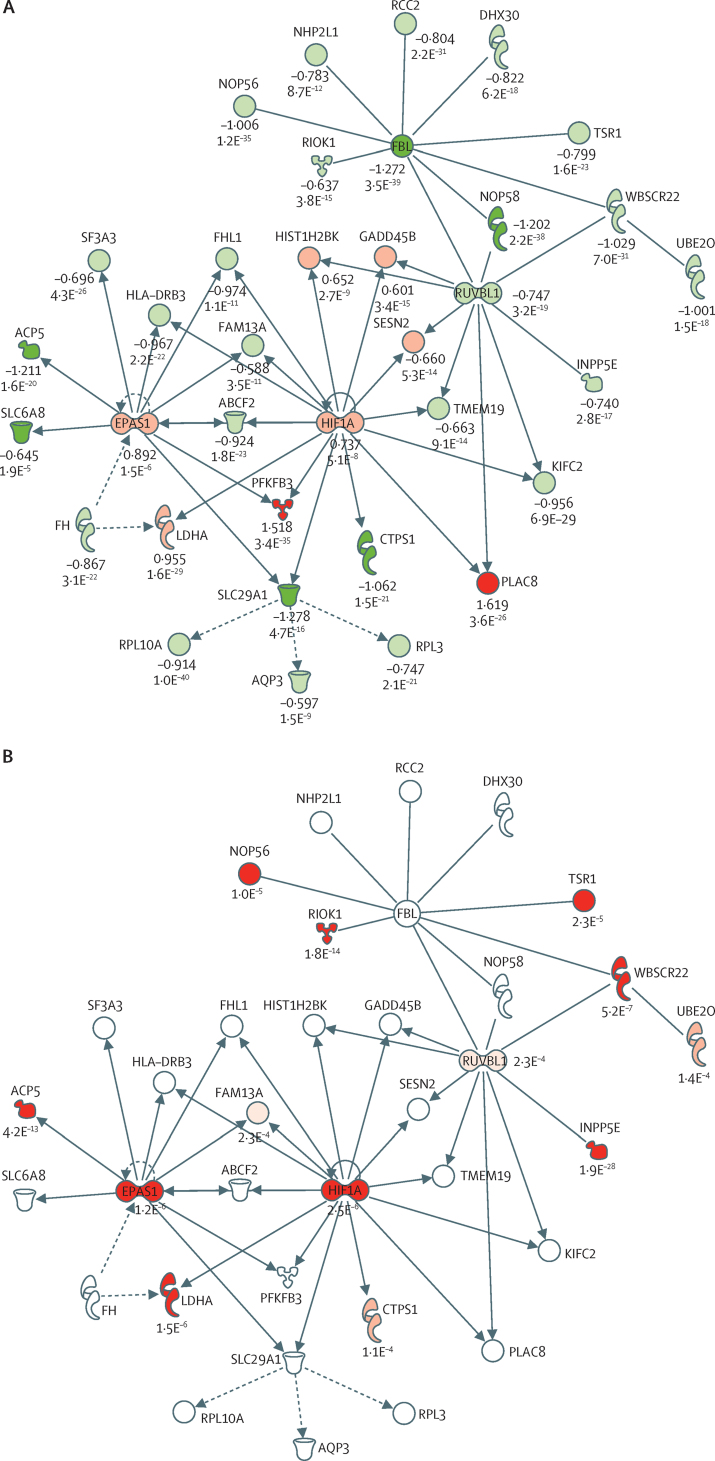
Differentially expressed networks between SRS The network with the lowest p value (p=1 × 10^−35^) identified from differential expression analysis of SRS1 versus SRS2 in the discovery cohort included 35 genes, with *HIF1A* and *EPAS1* as nodes (A). Log FC shown with FDR. Sepsis cis-eQTL affecting genes in hypoxia network (B). Presence of cis-eQTL shown by red molecules with p values. eQTL=expression quantitative trait loci. SRS=sepsis response signature. FC=fold change. FDR=false discovery rate.

We identified downregulation of human leucocyte antigen (HLA) class II genes and most genes implicated in T-cell activation in patients with SRS1 (figure 2, appendix 1 p 6). These downregulated genes included *CD247*, the main signal transduction element in the T-cell antigen receptor complex, and other gene signatures of T-cell exhaustion. Gene network analysis also showed differences associated with the hypoxic response, NF-κB, apoptosis, and histones (figure 3A, appendix 1 pp 7–9).

We next aimed to validate the SRS signature and observed relationship with outcome. We identified seven genes (*DYRK2, CCNB1IP1, TDRD9, ZAP70, ARL14EP, MDC1*, and *ADGRE3*) that were predictive of SRS group membership in the discovery cohort, with a misclassification rate of 3·8% as established with leave-one-out cross-validation. We then assigned group membership for the 106 patients in the validation cohort on the basis of expression of the seven gene set (table 1, appendix 2). Of these 106 patients, 37 (35%) were assigned SRS1 and 69 (65%) SRS2. In this validation cohort, we found that SRS group membership was again associated with outcome, in particular SRS1 with early mortality (14 day mortality was 59% for SRS1 *vs* 29% for SRS2, HR 2·8, 95% CI 1·5–5·1, p=0·0007; table 2, figure 1F), which supported our findings from the discovery cohort. On genome-wide analysis, we noted a strong correlation in differential gene expression between SRS groups in the discovery and validation cohorts (*r*^2^=0·82, p<2·2 × 10^−16^; appendix 1 p 10). Pathway analysis of differential gene expression similarly showed high concordance of enriched functions, including for gene signatures related to endotoxin tolerance and T-cell exhaustion, regulators, and gene networks (figure 1D, appendix 1 pp 6–9). We also detected a strong correlation (*r*^2^=0·84, p<2·2 × 10^−16^) between SRS groups in the validation cohort assigned with the seven gene signature and groups defined by hierarchical cluster analysis (appendix 1 p 11).

In view of our finding that gene expression signatures can define SRS groups that are informative for outcome, we compared this approach with models that use gene expression to predict individual mortality based on signatures generated with survival as a phenotype. We noted that models that used the most informative combinations of gene expression alone (misclassification rate 10·9% for 14 day survival and 16·2% for 28 day survival) performed better than did the most informative combinations of clinical covariates (misclassification rate 14% and 21·1%, respectively) in the discovery cohort (appendix 2). However when we tested the best predictive gene set for 14 day mortality in the validation cohort, the misclassification rate was high at 34% (appendix 2).

**Figure 4:**
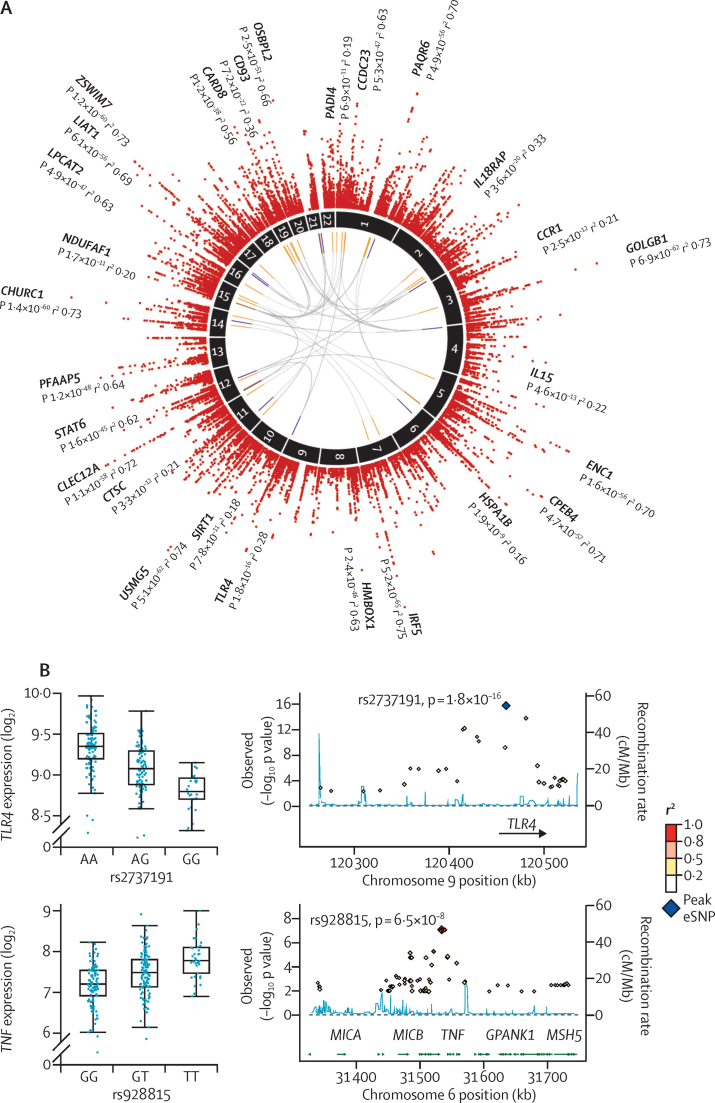
Sepsis eQTL Circos plot (A) of, from outer rim inwards, Manhattan plot for cis-eQTL in patients with sepsis (FDR <0·05) with names of genes with large effect sizes (variance explained, *r*^2^>70%) or examples of pathophysiological relevance shown; chromosome number; trans-eQTL (FDR<0·05) involving expression-associated SNPs also showing cis-eQTL (associated trans genes [orange] and SNPs [blue]). Box plots and local association plots for *TLR4* and *TNF* (B). eQTL=expression quantitative trait loci. SNP=single-nucleotide polymorphism. FDR=false discovery rate.

We then proceeded to investigate genomic modulators of sepsis. We proposed that individual heterogeneity in the transcriptomic response to sepsis might be modulated by genetic variation, and that such regulatory variants might only functionally modulate gene expression in the disease context of sepsis. To test this hypothesis, we mapped gene expression in sepsis as a quantitative trait for a subset of 240 patients in the discovery cohort. This analysis identified local, probably cis-acting, eQTL for 3795 genes (26·7% of those tested) with FDR less than 5% (2378 genes with FDR <1%; figure 4A, appendix 2). The median proportion of variance explained by the expression-associated SNP with the lowest p value for each probe was 9·4% (range 6·3–75·2), but this was more than 50% for immune-related genes such as *IRF5, CLEC12A, STAT6*, and *CARD8* (appendix 2).

Cis-eQTL genes (FDR <0·01) in patients with sepsis were significantly enriched for disease-relevant canonical pathways, including PI3K signalling, antigen presentation, and mitochondrial dysfunction (appendix 1 p 12, appendix 2). The predicted upstream regulators for the eQTL genes that we identified with the lowest p values were key mediators of the immune response to sepsis, including the death receptor FAS, transcription factor HNF4A, IFNγ, TNF, apoptotic regulator TP53, and cell death regulator BID (appendix 2). eQTL were most enriched in gene sets associated with viral respiratory infection and cellular immune response (appendix 1 p 13). These genes included cis-eQTL for *TLR4* and *TNF* (figure 4B) that we also previously reported in lipopolysaccharide-activated monocytes,^8^ as well as for *IFNAR2, TNFRSF10B, MAPK14, NFKB1, CASP7, IL15*, and *HGF*.

We next analysed trans-eQTL. We identified 171 associated genes (FDR <0·05; figure 4A, appendix 2) involving 380 SNPs, of which 42·6% of SNPs also show evidence of cis-eQTL (FDR <0·05). These eQTL included two known trans gene hubs proposed to be driven by local associations with lysozyme^19^ and the PAK1 inhibitor *CRIPAK*,^20^ in addition to a novel association with *VPS18,* which encodes a subunit of the HOPS tethering complex that is implicated in lysosome and endosome fusion and expression of genes located on six other chromosomes (appendix 1 p 14). We identified no overlap between the cis or trans-eQTL that we detected in sepsis and the association we previously found with sepsis outcome, which involved an intronic variant located in the *FER* gene.^6^

To further understand context specificity, we compared the cis-eQTL that we detected in sepsis with a meta-analysis of eQTL for whole blood from 5311 individuals.^17^ This comparison showed that cis-eQTL involving 1377 genes (36·3% of all genes with eQTL, FDR <0·05 in patients with sepsis) were seen only in sepsis (appendix 1 p 15). Such eQTL were located significantly more distally from the transcriptional start site than were those shared with individuals without sepsis (Mann-Whitney test p=9·4 × 10^−38^; appendix 1 p 16), consistent with context-specific eQTL being enriched at more distal regulatory elements.^8^, ^19^, ^21^

**Figure 5:**
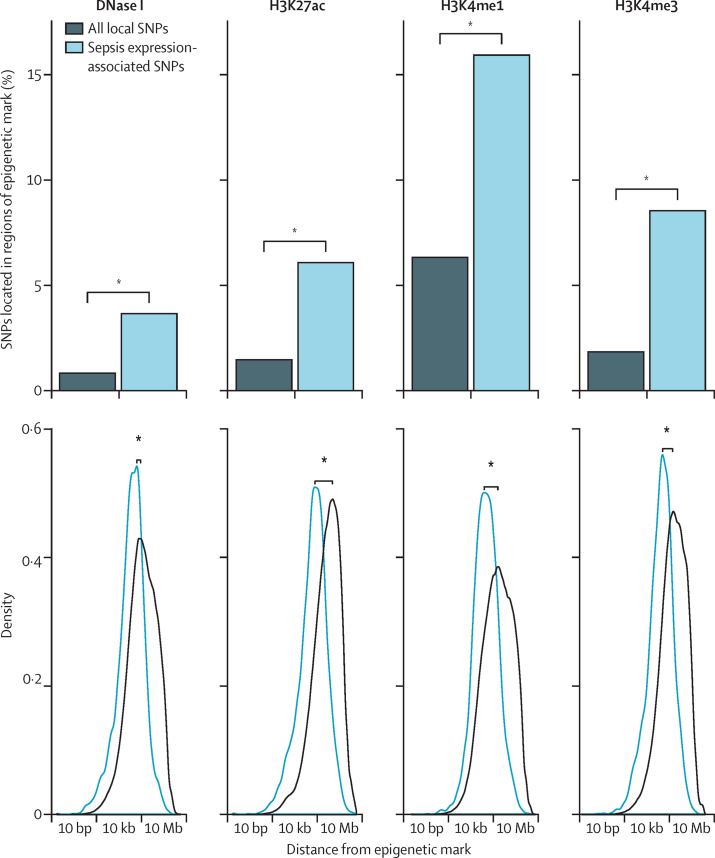
Sepsis eQTL and epigenetic marks Enrichment of expression-associated SNPs with the lowest p values for each gene (FDR<0·05) for sepsis cis-eQTL within DNase I hypersensitive sites (DNase I), and within peak intervals defined for different histone modifications (H3K27ac, H3K4me1, and H3K4me3), in monocytes tolerised by exposure to lipopolysaccharide for 24 h.^18^ p value shown for enrichment versus all local SNPs in terms of the proportion of expression-associated SNPs that overlapped with each mark (Fisher's exact test) and distance to the nearest mark (Mann-Whitney test). FDR=false discovery rate. SNP=single-nucleotide polymorphism. eQTL=expression quantitative trait loci. *p<2·2 × 10^−16^.

We next investigated whether sepsis eQTL were enriched in endotoxin tolerant genes and endotoxin-induced epigenetic marks. We found that sepsis cis-eQTL are enriched in the set of 331 endotoxin tolerant genes that we defined (χ^2^ p<0·0001). Expression-associated SNPs were significantly enriched in histone marks for active enhancer elements (H3K4me1 and H3K27ac) previously identified in lipopolysaccharide-treated monocytes^18^ compared with all SNPs within 1 Mb of a probe (2·91% *vs* 0·86%, p=2·02 × 10^−26^) and open chromatin predictive of regulatory sites (ie, DNase I hypersensitive sites; 3·65% *vs* 0·79%, p=1·98 × 10^−46^; figure 5). These results suggest that cis-eQTL in sepsis are often located in regulatory genomic elements specific to endotoxin-activated cells.

In the final phase of our analysis, we investigated the association between sepsis eQTL and SRS membership. In the genes differentially expressed between SRS groups, there was evidence of enrichment for eQTL (χ^2^ p<0·0001), including for genes previously implicated in the pathogenesis of sepsis and its treatment, such as *IL18RAP, CCR1, CCR3*, and *SIRT1* (appendix 1 pp 17–18 and appendix 2). Sepsis-associated eQTL were over-represented in specific pathways and networks enriched for genes that were differentially expressed between SRS groups (appendix 1 pp 7–8). The most significantly enriched gene network identified by differential gene expression analysis included hypoxia inducible transcription factors *HIF1A* (HIF1α) and *EPAS1* (HIF2α) as nodal genes (figure 3A, appendix 1 p 7). Both genes were upregulated in patients with SRS1 and each shows evidence of cis-eQTL in sepsis, together with 11 other genes in the network including lactate dehydrogenase A (*LDHA*; figure 3B). We identified significant enrichment of eQTL in this network (χ^2^ p=0·005).

**Figure 6:**
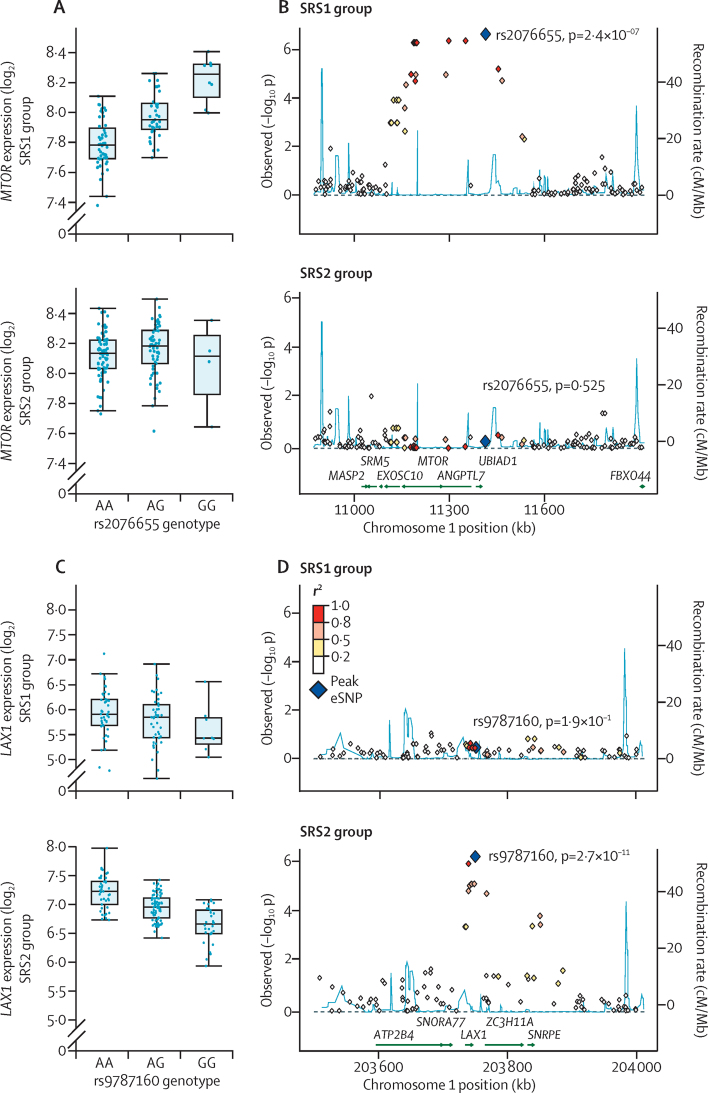
Sepsis eQTL and response state SRS1-specific eQTL for *MTOR* (A,B). There is reduced expression in SRS1 individuals (FC 0·85, FDR 4·9 × 10^−8^). SRS2-specific eQTL for *LAX1* (C,D)*. LAX1* is differentially expressed between SRS groups (0·44 FC, FDR 7·8 × 10^−25^). Box plots by allele for SRS1 and for SRS2 groups (A,C). Regional association plots (B,D). eQTL=expression quantitative trait loci. SRS=sepsis response signature. FC=fold change. FDR=false discovery rate.

We also discovered that *MTOR* was downregulated in patients with SRS1 and showed evidence of a sepsis eQTL. After mapping eQTL by SRS group, we noted that this association was only present in patients with SRS1 (figure 6A,B). We identified further examples of eQTL that were only significant in specific SRS groups (appendix 2), including for the heat shock transcription factor 2 gene *HSF2*, for which the eQTL was restricted to patients with SRS1 (appendix 1 pp 17–18), and genes such as *LAX1, TRIM44*, and *DDX24* for which eQTL were only detected in patients with SRS2 (figure 6C,D, appendix 2).

## Discussion

We propose that individual transcriptomic signatures that define the host response in sepsis will help to inform work establishing a precision medicine-based approach for sepsis management, refine the selection of patients for inclusion in clinical trials, and enable the development of novel targeted therapies. We found that for a critically ill population with sepsis caused by a specific source of infection (ie, community-acquired pneumonia), transcriptomic profiling of circulating peripheral blood leucocytes, which are central to the pathogenesis of sepsis, can define individual sepsis response signatures. One of these signatures (SRS1) identified a group of patients with features of relative immunosuppression, endotoxin tolerance, T-cell exhaustion, and metabolic derangement associated with a worse prognosis. Individuals with such a signature might be less able to eradicate infection and be susceptible to recurrent episodes of hospital-acquired infections and they might, therefore, benefit from therapy designed to boost their immune competence. Several such immune modulators are being advocated as potential treatments for sepsis, including cytokines (interleukin 7, interleukin 15, GM-CSF, interferon γ) and co-inhibitory molecular blockade (eg, anti-programmed cell death receptor-1 and anti-B and T lymphocyte attenuator).^22^

Our findings also support the idea that relative immunosuppression can occur soon after the onset of sepsis and contributes to early mortality. These results were supported by our showing in a separate cohort that the expression of seven genes is predictive of SRS group membership and early mortality. Moreover we found that clinical covariates, alone or in combination, did not predict SRS groupings and that our findings were robust to analyses that take into account differences in leucocyte cell count. Our results also demonstrate that the approach adopted by previous investigators of defining a gene expression signature predictive of individual mortality using survival to define groups^23^, ^24^ performed poorly in our replication cohort and is of little clinical utility. We propose that this poor performance reflects the many factors contributing to individual mortality in sepsis and contrasts with the ability of a gene expression signature to resolve a sepsis immune response state (as achieved with the SRS groups) that consistently associates with a worse prognosis.

Endotoxin tolerance is a crucial homoeostatic mechanism whereby initial endotoxin exposure results in a transient hyporesponsive state through epigenetic reprogramming and repression and induction of a set of genes. Our finding that patients with SRS1 have features of endotoxin tolerance based on transcriptomic signatures is consistent with previous evidence suggesting that dysregulation of endotoxin tolerance contributes to immunosuppression and mortality in sepsis.^25^, ^26^ Pena and colleagues,^26^ for example, reported that a gene expression signature of endotoxin tolerance derived ex vivo from peripheral blood mononuclear cells was predictive of the development of confirmed sepsis and organ dysfunction. Our finding that HLA class II genes are downregulated in patients with SRS1 is consistent with previous reports of associations between reduced class II expression and poor outcome in sepsis,^27^ while expression signatures of T-cell exhaustion are implicated in a recognised mechanism of immunosuppression in sepsis.^28^ We identified several predicted upstream regulators for the genes differentially expressed between SRS groups that are known to be involved in the pathogenesis of sepsis, including transglutaminase II,^29^ GM-CSF,^4^ and SATB1, which is crucial for chromatin remodelling and cytokine gene expression.^30^

We propose that the clustering of gene expression patterns and outcomes that we identified is likely to represent not only the premorbid condition of the host (eg, age, pre-existing comorbidities), severity of the infection (abundance or virulence of the infecting organism), and stage in the natural history of the illness,^4^ but might also be modulated by genomic sequence variation. Identification of eQTL in disease-relevant contexts is essential to understand the role of regulatory genetic variation in the host response given the context specificity of the action of such variants, especially in modulating the response to pathogens, as shown in research involving rhinovirus infection.^31^ We found evidence of sepsis eQTL that affected specific genes previously implicated in sepsis pathogenesis and treatment, including eQTL for *IL18RAP*, which encodes an accessory protein that enhances IL18-binding activity and is upregulated in patients with SRS1. *IL18R1* has been proposed as a biomarker of sepsis^32^ and neutralising IL18 signalling has been advocated as a therapeutic intervention.^33^ We also detected sepsis eQTL involving the most significant disease SNP for Behcet's disease,^34^ which we find modulates expression of the chemokine receptor genes *CCR1* and *CCR3*, which are upregulated and downregulated, respectively, in patients with SRS1. Antagonists to CCR1 attenuate the systemic inflammatory response in sepsis^35^ and CCR3 expression is reduced in septic shock.^36^ Other examples of sepsis eQTL that we identified and were relevant to sepsis pathogenesis included *MLKL* (necroptosis), *NLRC5* (regulation of the major histocompatibility complex), *HSPA1B* (heat shock), *IER3* (protection from apoptosis^37^), *CTSC* (granule-mediated apoptosis and antiviral protection^38^), and *PADI4* (initiates neutrophil NETosis in sepsis, promoting host defence^39^).

Our pathway analysis identified genetic variants associated with the expression of genes implicated in key processes in the immune and metabolic response to infection. For example, our detection of eQTL for *HIF1A, EPAS1*, and *MTOR* shows how individual predisposition could modulate the metabolic basis of trained immunity. HIF1α is central to the functional reprogramming of monocytes in sepsis whereby tolerance, tissue remodelling, and antimicrobial responses are controlled,^40^ while epigenetic reprogramming dependent on the Akt–mTOR–HIF1α pathway modulates an effective immune response to sepsis in mice.^41^ mTOR is crucial in the switch to pro-inflammatory glycolysis that occurs in sepsis.^41^ Similarly, we also identified cis-eQTL for *LDHA*, which is responsible for the final step in anaerobic glycolysis and was significantly upregulated in patients with SRS1. We previously found that this eQTL is also present in monocytes but is specific to cells treated with lipopolysaccharide for 24 h.^8^ Serum lactate is an important clinical biomarker associated with organ failure and mortality in sepsis.^42^ We also found that *GAPDH*, which encodes an enzyme required for glycolysis and is linked to sepsis-related acute lung injury,^43^ was significantly upregulated, consistent with a metabolic switch from oxidative phosphorylation to glycolysis in patients with SRS1,^41^ but with no evidence of an eQTL. Furthermore, *SIRT1*, which encodes a histone deacetylase essential to nuclear–mitochondrial communication during immune–metabolic adaptation in sepsis,^44^ and which modulates epigenetic gene silencing in endotoxin tolerance, was downregulated in patients with SRS1 and has a sepsis cis-eQTL.

The cis-eQTL that we identified for *MTOR* was only present in patients with SRS1. Other examples of such eQTL included *HSF2*, which encodes heat shock transcription factor 2, which is crucial to regulation of the heat shock response in infection, inflammation, and oxidative stress and has evidence of association with mortality in sepsis.^45^ By contrast, *LAX1*, which encodes a negative regulator of T-cell activation,^46^ showed evidence of eQTL only in patients with SRS2; in this case, the lead expression-associated SNP is also an induced eQTL in lipopolysaccharide-stimulated monocytes.^8^ SRS2 group eQTL specificity was also seen for *TRIM44*, which promotes an enhanced cellular response to viral infection,^47^ and *DDX24*, which encodes a type I interferon-inducible DEAD-box protein RNA-helicase modulating IRF7 activity.^48^

There are necessarily limitations to this study. We recognise that further work is needed to understand the temporal, cellular, and tissue specificities of our findings. It will also be important to establish whether the same SRS can be found in patients with sepsis of different causes. Other investigators have reported the potential clinical utility of gene expression profiling at ICU admission, showing that the ratio of expression of two negative regulators of apoptosis (*FAIM3* and *PLAC8*) could be used to assist in the rapid diagnosis of community-acquired pneumonia,^49^ while in paediatric patients with septic shock, transcriptomics might be useful for patient subclassification and identification of individuals who might benefit from adjunctive corticosteroids.^50^ Proteomic analysis would help to further validate our transcriptomic findings and more research is needed at an immunological level to functionally characterise the SRS phenotypes. Our finding that eQTL in patients with sepsis affect key mediators of disease requires further functional characterisation to establish causal mechanistic associations for specific genetic variants and to establish their physiological consequences. For trans effects at a distance, larger sample sizes will be needed to provide sufficient power to find additional moderate-to-small genetic effects.^17^ We did not find overlap with our previous genome-wide association study (GWAS) of sepsis that had implicated the *FER* gene in sepsis outcome.^6^ However we noted that this gene was expressed at low levels in the total leucocyte population, and the disease-associated variant might act through effects on splicing or other mechanisms. To aid understanding of the role of regulatory genetic variants in sepsis susceptibility and disease outcome, we suggest that further GWAS will be needed in which the disease phenotype is more precisely defined, for example based on SRS group.

In view of the persistently high mortality associated with sepsis and the absence of specific therapies,^2^ advances based on modulating the septic response are needed urgently. Our analysis of interindividual variation in transcriptomic responses in a disease context shows that this approach can provide insights into the taxonomy of acute illness by improved phenotypic classification of sepsis patients. Improved classification could have potentially important therapeutic implications, including for the development of novel interventions, identification of high risk patients, and individually targeted therapy aiming to modulate the dysfunctional host response.

## Figures and Tables

**Table 1:** Characteristics of patients included in the discovery and validation cohorts

		**Discovery cohort (n=265)**	**Validation cohort (n=106)**
Age (years)		62 (16)	69 (14)
Male sex		145 (55%)	79 (75%)
APACHE II score		18 (7)	23 (9)
SOFA score		6 (4)	7 (4)
Mortality			
	14 day	40 (15%)	42 (40%)
	28 day	56 (21%)	52 (49%)
	6 month	78 (29%)	56 (53%)
Infection			
	Gram-positive bacteria	47 (18%)	13 (12%)
	Gram-negative bacteria	25 (9%)	10 (9%)
	Viral	25 (9%)	3 (3%)
Mechanical ventilation		191 (72%)	82 (77%)
Vasopressors		107 (40%)	46 (43%)

Data are n (%) or mean (SD) unless otherwise specified. APACHE II=Acute Physiology and Chronic Health Evaluation II. SOFA=Sequential Organ Failure Assessment on day of sampling.

**Table 2:** Characteristics of patients included in the discovery and validation cohorts by SRS groups

		**Discovery cohort**			**Validation cohort**		
		SRS group 1 (n=108)	SRS group 2 (n=157)	p value	SRS group 1 (n=37)	SRS group 2 (n=69)	p value
Age (years)		60·2 (15·3)	62·9 (16·6)	0·18	67·8 (15·0)	69·8 (14·1)	0·52
Male sex		60 (56%)	85 (54%)	0·92*	25 (68%)	54 (78%)	0·33*
APACHE II score		19 (6·9)	18 (6·3)	0·24	25·1 (9·3)	21·3 (7·9)	0·04
SOFA score		7·9 (4·0)	5·4 (3·2)	2·1 × 10^−7^	8·6 (4·9)	6·7 (3·6)	0·033
Arterial pressure (lowest, mm Hg)		63·5 (10·5)	70·1 (13·6)	1·8 × 10^−5^	63·1 (13·2)	68·6 (10·7)	0·03
Mechanical ventilation		82 (76%)	109 (69%)	0·31*	30 (81%)	52 (75%)	0·67*
Vasopressors/inotropes				0·001*			0·07*
	No dose	50 (46%)	108 (69%)		18 (49%)	42 (61%)	
	Low dose	1 (1%)	0		0	1 (<1%)	
	Medium dose	17 (16%)	10 (6%)		2 (5%)	10 (14%)	
	High dose	40 (37%)	39 (25%)		17 (46%)	16 (23%)	
Bicarbonate (mmol/L)		23 (15·9)	27·6 (5·6)	6·9 × 10^−9^	21·5 (6·4)	25·4 (6·7)	0·005
Renal replacement therapy		17 (16%)	14 (9%)	0·13*	10 (27%)	6 (9%)	0·03*
Proportion of leucocytes							
	Polynucleocytes	0·89 (0·07)	0·82 (0·09)	9·6 × 10^−12^	0·88 (0·12)	0·83 (0·10)	0·03
	Lymphocytes	0·06 (0·05)	0·11 (0·07)	7·2 × 10^−10^	0·06 (0·06)	0·10 (0·06)	0·004
	Mononucleocytes	0·05 (0·04)	0·08 (0·05)	1·5 × 10^−5^	0·06 (0·09)	0·07 (0·06)	0·36
Platelets (×10^3^ cells per mL)		182·8 (91·5)	246·8 (119·4)	1·4 × 10^−6^	181·1 (98·7)	224·0 (105·8)	0·04
Infection							
	Gram-positive bacteria	24 (22%)	23 (15%)	0·16*	7 (19%)	6 (9%)	0·22*
	Gram-negative bacteria	12 (11%)	13 (8%)	0·58*	6 (16%)	4 (6%)	0·16*
	Viral	8 (7%)	17 (11%)	0·48*	0	3 (4%)	0·50*
Mortality							
	14 day	24 (22%)	16 (10%)	0·005†	22 (59%)	20 (29%)	0·0007†
	28 day	29 (27%)	27 (17%)	0·037†	24 (65%)	28 (41%)	0·003†
	6 month	39 (36%)	39 (25%)	0·032†	25 (68%)	31 (45%)	0·004†

Data are n (%) or mean (SD) unless otherwise specified. APACHE II=Acute Physiology and Chronic Health Evaluation II. SOFA=Sequential Organ Failure Assessment (score on day of sampling). Statistical analysis *t* test unless otherwise specified.

* χ^2^ test.

† Log-rank test. SRS=sepsis response signature.
