# Supplementary material for: Genomic landscape of the individual host response and outcomes in sepsis: a prospective cohort study
Source: Lancet Respir Med. 2016 Apr;4(4):259–71. doi: 10.1016/S2213-2600(16)00046-1 (PMC4820667; doi:10.1016/S2213-2600(16)00046-1)
Supplement: Supplementary appendix 1 [file mmc1.pdf]

THE LANCET
Respiratory Medicine

Supplementary appendix 1

This appendix formed part of the original submission and has been peer reviewed. We post it as supplied by the authors.

Supplement to: Davenport EE, Burnham KL, Radhakrishnan J, et al. Genomic landscape of the individual host response and outcomes in sepsis: a prospective cohort study. *Lancet Respir Med* 2016; published online Feb 22. http://dx.doi.org/10.1016/S2213-2600(16)00046-1.

Appendix

Genomic landscape of the individual host response and outcomes in sepsis: a prospective cohort study

Davenport et al

Overview of content

Supplementary Methods

Supplementary References

Supplementary Figures

Figure S1. Principal components and eQTL mapping.

Figure S2. MHC gene expression and T cell activation among differentially expressed genes between patient SRS groups.

Figure S3. Hypoxia related network among differentially expressed genes between patient SRS groups.

Figure S4. NF-κB related network among differentially expressed genes between patient SRS groups.

Figure S5. MYC and histone related networks identified among differentially expressed genes between patient SRS groups.

Figure S6. Correlation of differential gene expression between SRS groups in derivation and validation cohorts.

Figure S7. Correlation of differential gene expression between groups in the validation cohort determined using the SRS predictive gene set or an unsupervised clustering approach.

Figure S8. PI3K signalling canonical pathway enrichment for sepsis cis-eQTL.

Figure S9. Pathway enrichment for sepsis cis-eQTL (FDR <0.01).

Figure S10. Trans-eQTL gene hub involving rs12148454.

Figure S11. Sepsis-specific cis-eQTL.

Figure S12. eQTL shared or specific to sepsis and distance from the transcription start site (TSS).

Figure S13. Sepsis cis-eQTL involving genes differentially expressed between SRS groups and SRS-specific eQTL.

Figure S14. MDS plots comparing the genetic ancestry of sepsis (GAinS) patients to 1000 Genomes Project populations.

Supplementary Tables

Table S1 Participating hospitals involved in patient recruitment and GAinS Investigators

Supplementary Methods

Case definitions and phenotyping

The diagnosis of sepsis was based on the International Consensus Criteria (2003)[1] with all patients reported here showing some degree of organ dysfunction during ICU admission.[2] Community acquired pneumonia was defined as a febrile illness associated with cough, sputum production, breathlessness, leucocytosis and radiological features of pneumonia acquired in the community or within less than 2 days of hospital admission.[3,4] Exclusion criteria were: patient or legal representative unwilling or unable to give consent; patient <18 years of age; pregnancy; an advanced directive to withhold or withdraw life sustaining treatment; admission for palliative care only; or immune-compromise. Ethics approval was granted nationally (REC Reference Number 05/MRE00/38 and 08/H0505/78) and for individual participating centres. Written, informed consent was obtained from all patients or a legal representative. An electronic case report form (eCRF) was used to record demographics and clinical covariates. Microbiological investigations were performed according to local policies and practices with organism(s) isolated, source and the use of serological methods recorded in the eCRF. Patients were followed up for 6 months following ICU discharge with date of death recorded.

Sample collection

Samples for RNA were obtained after ICU admission (at the time of study enrolment, a window up to day 5) by collecting 5 ml blood into Vacuette EDTA tubes (Becton Dickinson). The total blood leucocyte population was isolated on the ICU using the LeukoLOCK filter system (Ambion).[5] Blood was passed across the leucocyte enrichment filter using a vacutainer system. The filtered leucocytes were stabilised with RNAlater. In addition, whole blood was collected for DNA extraction.

RNA extraction

Purified RNA, depleted of globin mRNA, was extracted from the LeukoLOCK filters using the Total RNA Isolation Protocol (Ambion). The contents of the filter were lysed and eluted with a guanidine thiocyanate-based solution. Cellular proteins and DNA were degraded using Proteinase K and DNase I respectively. The RNA was then purified using magnetic bead technology. Spectrophotometry (Nanodrop 2000; Thermo Scientific) was used to quantitate the RNA yield and the quality of a small subset was determined by on-chip electrophoresis (Biorad Bioanalyzer; Agilent).

Genomic DNA extraction

DNA was extracted from the buffy layer (or when not available, whole blood) using either the Qiagen DNA extraction protocol, the automated Maxwell 16 Blood purification kit (Promega) or the QIAamp Blood Midi kit protocol (Qiagen). The DNA yield was determined by fluorescence using the Quant-iT PicoGreen kit (Invitrogen).

Microarray data processing, pathway analysis and hierarchical cluster analysis

Genome-wide gene expression analysis was carried out on 1000ng RNA using the Illumina Human-HT-12 v4 Expression BeadChip gene expression platform comprising 47,231 probes. The report for analysis was generated by Illumina's Genomestudio software. Data backgrounds were subtracted and probes that did not have a detection value greater than or equal to 0.95 in at least 5% of samples were removed. The raw data were transformed and normalised using the Variance Stabilisation and Normalisation method.[6] Quality control (QC) checks including principal component analysis (PCA) to identify batch and array effects were carried out using R.[7] Following QC, five samples were removed from the discovery cohort and eight samples from the validation cohort. In addition, in order to assign SRS using the gene expression model, the raw validation data were restricted to probes retained in the original cohort and normalised against the discovery data. Pre-processing, QC checks and statistical analyses were conducted separately for the discovery and validation datasets.

We identified the optimal number of sepsis response signature (SRS) groups (2-4) using within-group sum of squares. Significant associations with early mortality (14-day survival) remained when the number of groups was increased to 3 (P value 0.036) or 4 (P value 0.0015). We therefore selected the minimum number of groups that explained the difference in survival in order to maximize power to detect phenotypic differences between groups.

For a false discovery rate of 0.05 with 3000 genes differentially expressed between SRS groups, the power to detect a 1.5-fold difference in expression between SRS1 and SRS2 with a minimal sample size of 37 patients in one group will be 1. To select predictive gene sets, the data were first restricted to genes with moderate to high expression (log_2(expression) >6.5 in a proportion of samples which equated to the smallest group of a comparison) and showed >2 fold change (FC) between SRS groups or >1.5FC between survivors and non-

survivors. Models with a small number of predictors were then generated using a methodology developed for data sets with many more variables than observations, which fits response models with a sparsity prior and carries out simultaneous variable selection and parameter estimation.[8] R packages[7] used included limma[9] (differential gene expression), FactoMineR[10] (hierarchical clustering) and GeneRave CSIRO Bioinformatics version 3.0.8[8] (to identify clinical covariates or genes for use in prediction models).

Genotyping

Genotyping was performed for 730,525 SNPs using the Illumina HumanOmniExpress BeadChip. PLINK[11] was used for the genotyping QC. Sample QC included discordant sex information, proportion of missing genotypes (>0.02), heterozygosity rate, identity by descent (pi hat >0.1875) and multi-dimensional scaling with 1000 Genomes populations (Figure S14). SNP QC included SNP missing data proportion (>0.02), minor allele frequency (MAF) (<0.01) and HWE ($<1x10^{-10}$). For both the SRS cluster specific and trans-eQTL analysis, SNPs with MAF < 0.05 were excluded.

eQTL analysis

Following QC, 240 patients within the discovery cohort had good quality genome-wide gene expression and genotyping data for eQTL analysis. We conducted two further QC steps at the probe level to reduce potential confounding effects. Firstly, we excluded probes with sequences that mapped to more than one genomic location using BLAST. Secondly, we excluded probes corresponding to sequences with a SNP present at a MAF of at least 1% in European populations (1000 Genomes Project). eQTL analysis was performed using an additive linear model by the R package Matrix eQTL[12]. We included major principal components (PCs) of the gene expression data as covariates in the eQTL analysis to limit the effect of confounding factors. As noted by other investigators mapping eQTL in different contexts, this enhances detection of eQTLs[13-15]. The number of PCs to include was determined by mapping cis eQTL with increasing numbers of PCs to maximise the number of unique probes with cis eQTL associations. We included the first 30 PCs for the main eQTL (Figure S1) and the first 25 PCs for the individual SRS group eQTL.

Comparison with whole blood eQTL meta-analysis

Sepsis eQTL were compared to the Westra et al eQTL meta-analysis dataset.[15] We restricted the analysis to the 13,590 genes shared across the Illumina Expression BeadChips used and compared the datasets for shared and specific eQTL at the gene level. eQTL were considered likely to be sepsis-specific if no significant association (FDR <0.05) was seen for a given gene in the Westra data.

Annotation of eQTL with epigenomic features

Following exclusion of Y chromosome SNPs, 3,643 unique peak eSNPs showing evidence of sepsis cis-eQTL (FDR<0.05) were annotated with chromatin features for lipopolysaccharide stimulated monocytes from the Blueprint Consortium[16] downloaded from their ftp server. The data included histone marks (H3K27ac, H3K4me1 and H3K4me3) for two samples and DNase I hypersensitivity for four samples. Data were available for all autosomes and the X chromosome. Consensus peaks were defined by presence in at least two samples. The proportion of eSNPs that overlapped with each mark was compared to the proportion for SNPs within 1Mb of a probe (n=614,176) by Fisher's Exact test, and distance to the nearest mark by Mann-Whitney test.

Endotoxin tolerance

Two human endotoxin tolerance datasets were accessed through NCBI GEO database (http://www.ncbi.nlm.nih.gov/geo/), accession GSE15219 and GSE22248. The transcriptomic response to a single lipopolysaccharide treatment was first established. The expression of these lipopolysaccharide response genes in cells stimulated once was then compared to their expression in cells from the same subject that had been treated twice to identify differentially responding genes. Combining these two studies defined an endotoxin tolerance gene signature comprising 398 genes (FDR <0.05, FC >1.5), of which 331 were measured in the sepsis patients. Enrichment of this tolerance signature within the genes differentially expressed between SRS groups was assessed using ROAST[17], a rotation-based gene set test that takes account of directionality in addition to significance, enabling the likelihood of the SRS1 group expression signature being enriched for the endotoxin tolerance signature to be determined.

References

1. Levy MM, Fink MP, Marshall JC, et al. 2001 SCCM/ESICM/ACCP/ATS/SIS International Sepsis Definitions Conference. *Intensive Care Med* 2003; **29**(4): 530-8.
2. Vincent JL, Opal SM, Marshall JC, Tracey KJ. Sepsis definitions: time for change. *Lancet* 2013; **381**(9868): 774-5.
3. Angus DC, Marrie TJ, Obrosky DS, et al. Severe community-acquired pneumonia: use of intensive care services and evaluation of American and British Thoracic Society Diagnostic criteria. *Am J Respir Crit Care Med* 2002; **166**(5): 717-23.
4. Walden AP, Clarke GM, McKechnie S, et al. Patients with community acquired pneumonia admitted to European intensive care units: an epidemiological survey of the GenOSept cohort. *Crit Care* 2014; **18**(2): R58.
5. Idaghdour Y, Storey JD, Jadallah SJ, Gibson G. A genome-wide gene expression signature of environmental geography in leukocytes of Moroccan Amazighs. *PLoS Genet* 2008; **4**(4): e1000052.
6. Huber W, von Heydebreck A, Sultmann H, Poustka A, Vingron M. Variance stabilization applied to microarray data calibration and to the quantification of differential expression. *Bioinformatics* 2002; **18 Suppl 1**: S96-104.
7. R Core Team. R: A language and environment for statistical computing (R Foundation for Statistical Computing, Vienna, Austria). url: http://www.Rproject.org/. 2013.
8. Kiiveri HT. A general approach to simultaneous model fitting and variable elimination in response models for biological data with many more variables than observations. *BMC Bioinformatics* 2008; **9**: 195.
9. Smyth GK. Linear models and empirical bayes methods for assessing differential expression in microarray experiments. *Stat Appl Genet Mol Biol* 2004; **3**: Article3.
10. Husson FJ, Josse J, Le S, Mazet J. FactoMineR: Multivariate Exploratory Data Analysis and Data Mining with R. 2013.
11. Purcell S, Neale B, Todd-Brown K, et al. PLINK: a tool set for whole-genome association and population-based linkage analyses. *Am J Hum Genet* 2007; **81**(3): 559-75.
12. Shabalin AA. Matrix eQTL: ultra fast eQTL analysis via large matrix operations. *Bioinformatics* 2012; **28**(10): 1353-8.
13. Biswas S, Storey JD, Akey JM. Mapping gene expression quantitative trait loci by singular value decomposition and independent component analysis. *BMC Bioinformatics* 2008; **9**: 244.
14. Fehrmann RS, Jansen RC, Veldink JH, et al. Trans-eQTLs Reveal That Independent Genetic Variants Associated with a Complex Phenotype Converge on Intermediate Genes, with a Major Role for the HLA. *PLoS Genet* 2011; **7**(8): e1002197.
15. Westra HJ, Peters MJ, Esko T, et al. Systematic identification of trans eQTLs as putative drivers of known disease associations. *Nat Genet* 2013; **45**: 1238-43.
16. Saeed S, Quintin J, Kerstens HH, et al. Epigenetic programming of monocyte-to-macrophage differentiation and trained innate immunity. *Science* 2014; **345**(6204): 1251086.
17. Wu D, Lim E, Vaillant F, Asselin-Labat ML, Visvader JE, Smyth GK. ROAST: rotation gene set tests for complex microarray experiments. *Bioinformatics* 2010; **26**(17): 2176-82.
18. Hotchkiss RS, Swanson PE, Knudson CM, et al. Overexpression of Bcl-2 in transgenic mice decreases apoptosis and improves survival in sepsis. *J Immunol* 1999; **162**(7): 4148-56.
19. Zhang DW, Shao J, Lin J, et al. RIP3, an energy metabolism regulator that switches TNF-induced cell death from apoptosis to necrosis. *Science* 2009; **325**(5938): 332-6.
20. Rodrigue-Gervais IG, Labbe K, Dagenais M, et al. Cellular inhibitor of apoptosis protein cIAP2 protects against pulmonary tissue necrosis during influenza virus infection to promote host survival. *Cell Host Microbe* 2014; **15**(1): 23-35.
21. Allam R, Kumar SV, Darisipudi MN, Anders HJ. Extracellular histones in tissue injury and inflammation. *J Mol Med* 2014; **92**(5): 465-72.
22. Liberali P, Snijder B, Pelkmans L. A hierarchical map of regulatory genetic interactions in membrane trafficking. *Cell* 2014; **157**(6): 1473-87.

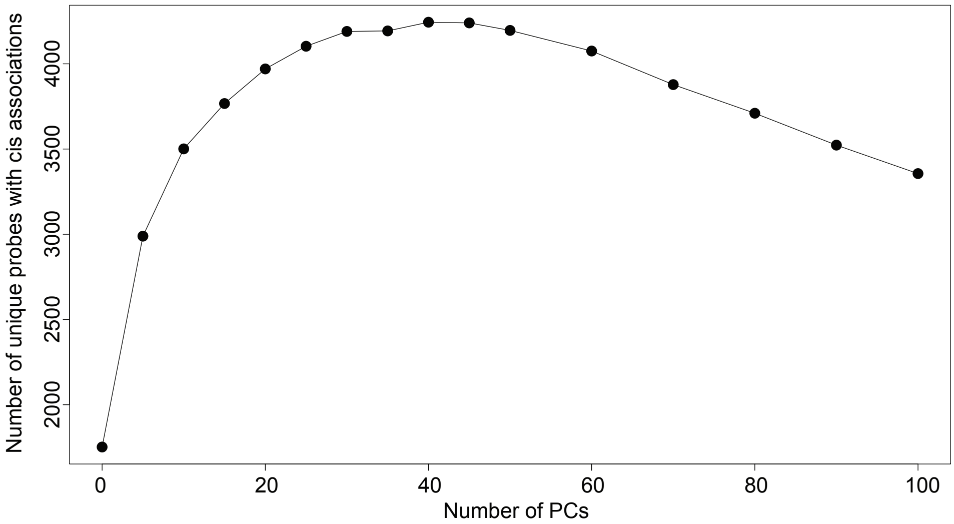

Figure S1. Principal components and eQTL mapping. Analysis of the effects of incorporation of major associated principal components (PC) of the expression data as covariates on observed cis-eQTL. PCs correlated with genotype were not used. The number of unique probes with a cis association is plotted.

A

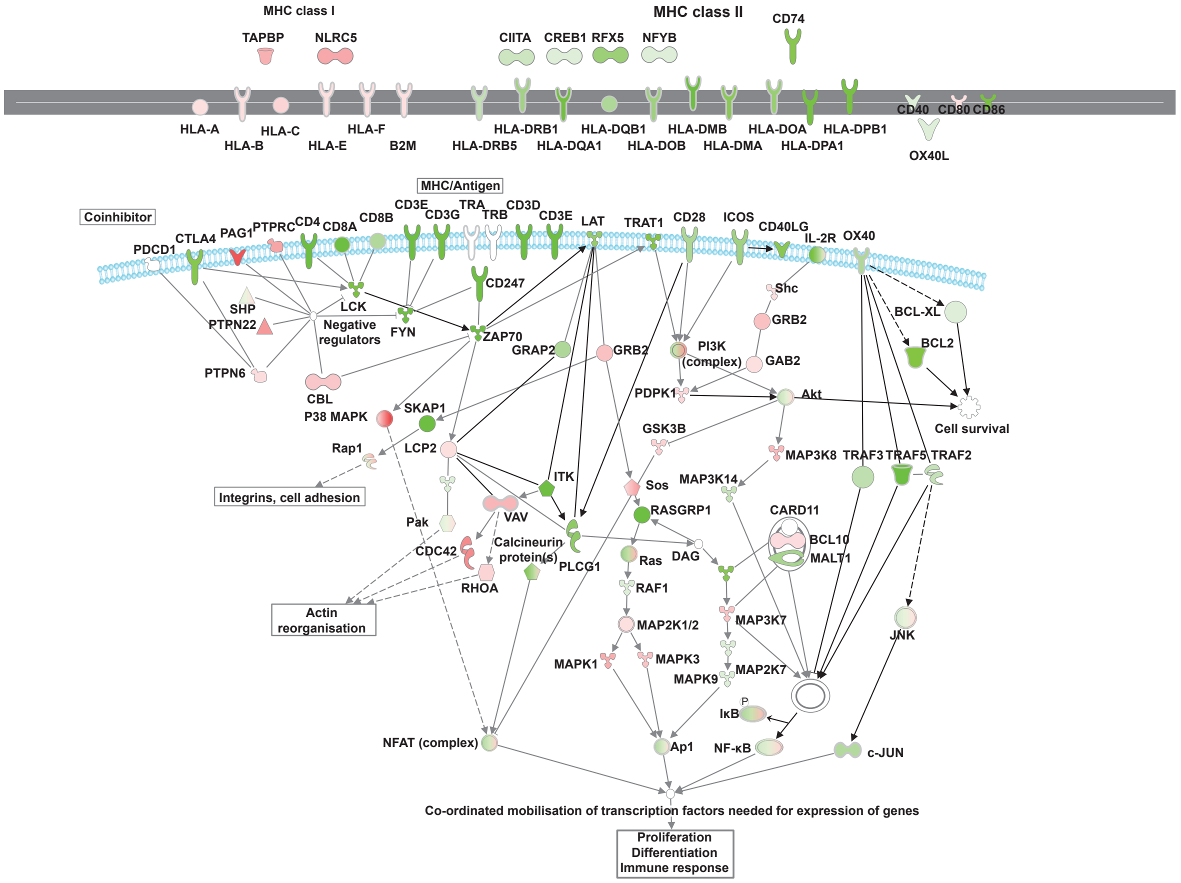

B

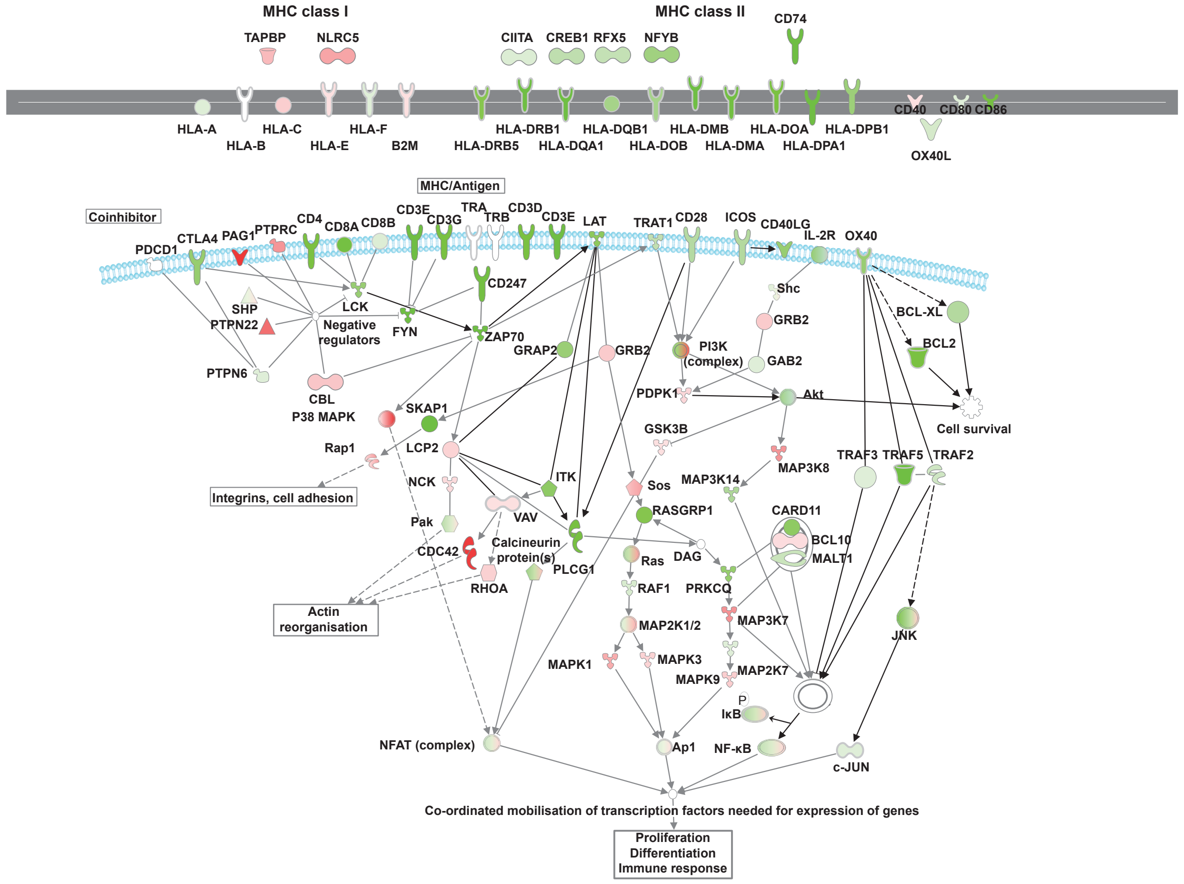

Figure S2. MHC gene expression and T cell activation among differentially expressed genes between patient SRS groups. Differentially expressed genes (FC >1.5, FDR <0.05) between patients in groups SRS1 and 2 involving MHC and T cell activation are shown for **(A)** the discovery cohort (n=265) and **(B)** the validation cohort (n=106) with red shading indicating up-regulation, green shading down-regulation (relative to the SRS1 group). Pathways derived from manual annotation and Kegg pathway hsa04660.

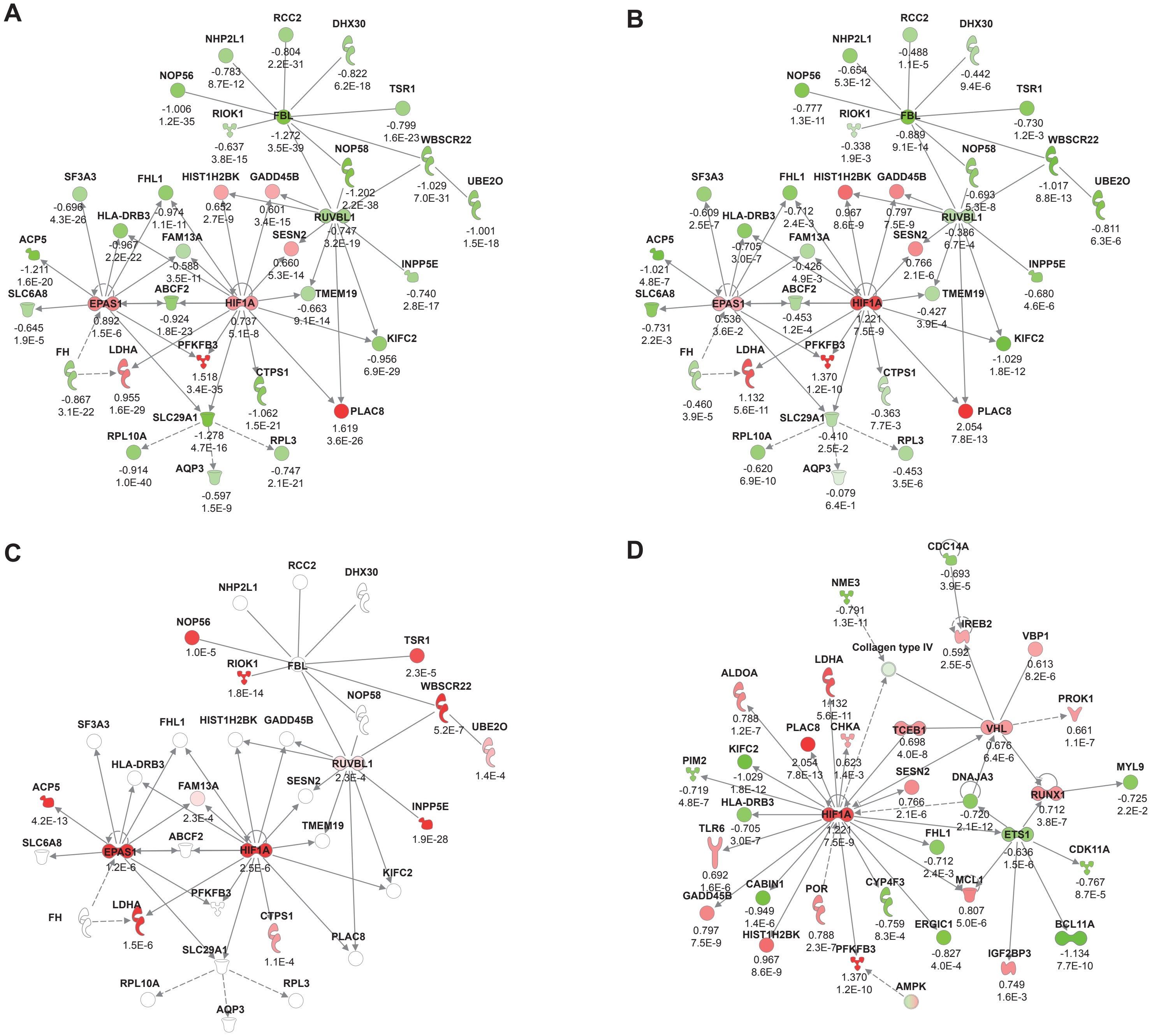

Figure S3. Hypoxia related network among differentially expressed genes between patient SRS groups. (**A**) The most significant network (P 1x10^{-35}, indicating likelihood of the genes in a network being found together due to random chance) identified on network analysis of differentially expressed genes between SRS groups 1 and 2 in the discovery cohort included 35 genes of which *HIF1A* (HIF1α) and *EPAS1* (HIF2α) are nodal genes. Log fold change is shown below molecular symbols together with FDR. (**B**) Gene expression data for differentially expressed genes in the validation cohort overlaid on the same gene network. Log fold change is shown below molecular symbols together with FDR. (**C**) Sepsis cis-eQTL involving genes in network (where cis-eQTL are found, molecules are shaded and P values shown). (**D**) In the validation cohort, the second most significant network identified (P 1x10^{-28}) was also related to hypoxia with *HIF1A* as a nodal gene and included 9 other genes shared with (a). Log fold change is shown below molecular symbols together with FDR.

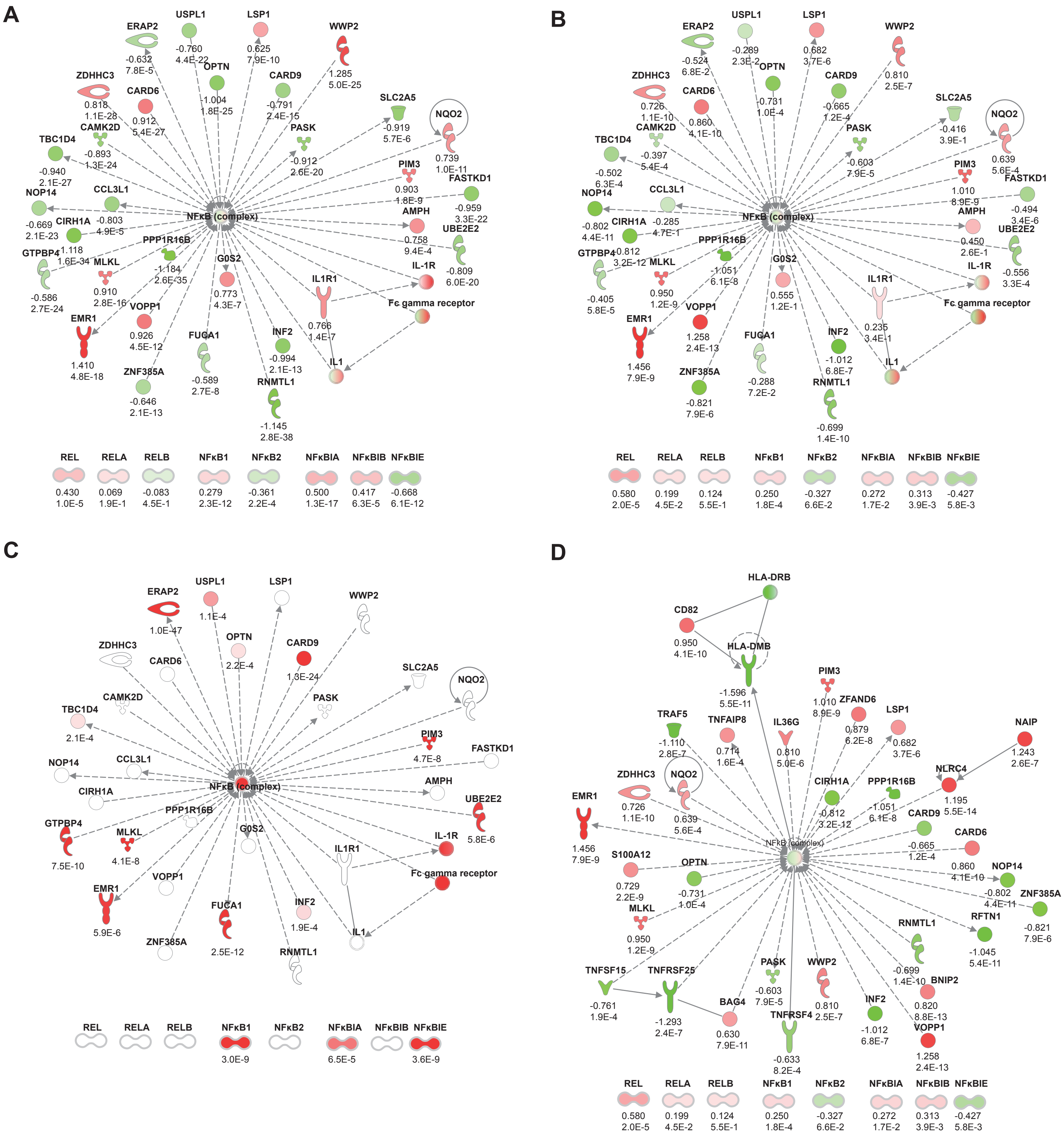

Figure S4. NF-κB related network among differentially expressed genes between patient SRS groups. (**A**) Gene network identified on network analysis of differentially expressed genes between SRS groups 1 and 2 in the discovery cohort included 31 genes with NF-κB complex as the node (individual NF-κB genes shown below nodal network) (P $1x10^{-25}$). Log fold change is shown below molecular symbols together with FDR. (**B**) Gene expression data for differentially expressed genes in the validation cohort overlaid on the same gene network. Log fold change is shown below molecular symbols together with FDR. (**C**) Sepsis cis-eQTL involving genes in discovery data network (where cis-eQTL are found, molecules are shaded and P values shown). (**D**) In the validation cohort, the most significant network identified was also related to NF-κB (P $1x10^{-28}$) involving 33 genes. Log fold change is shown below molecular symbols together with FDR.

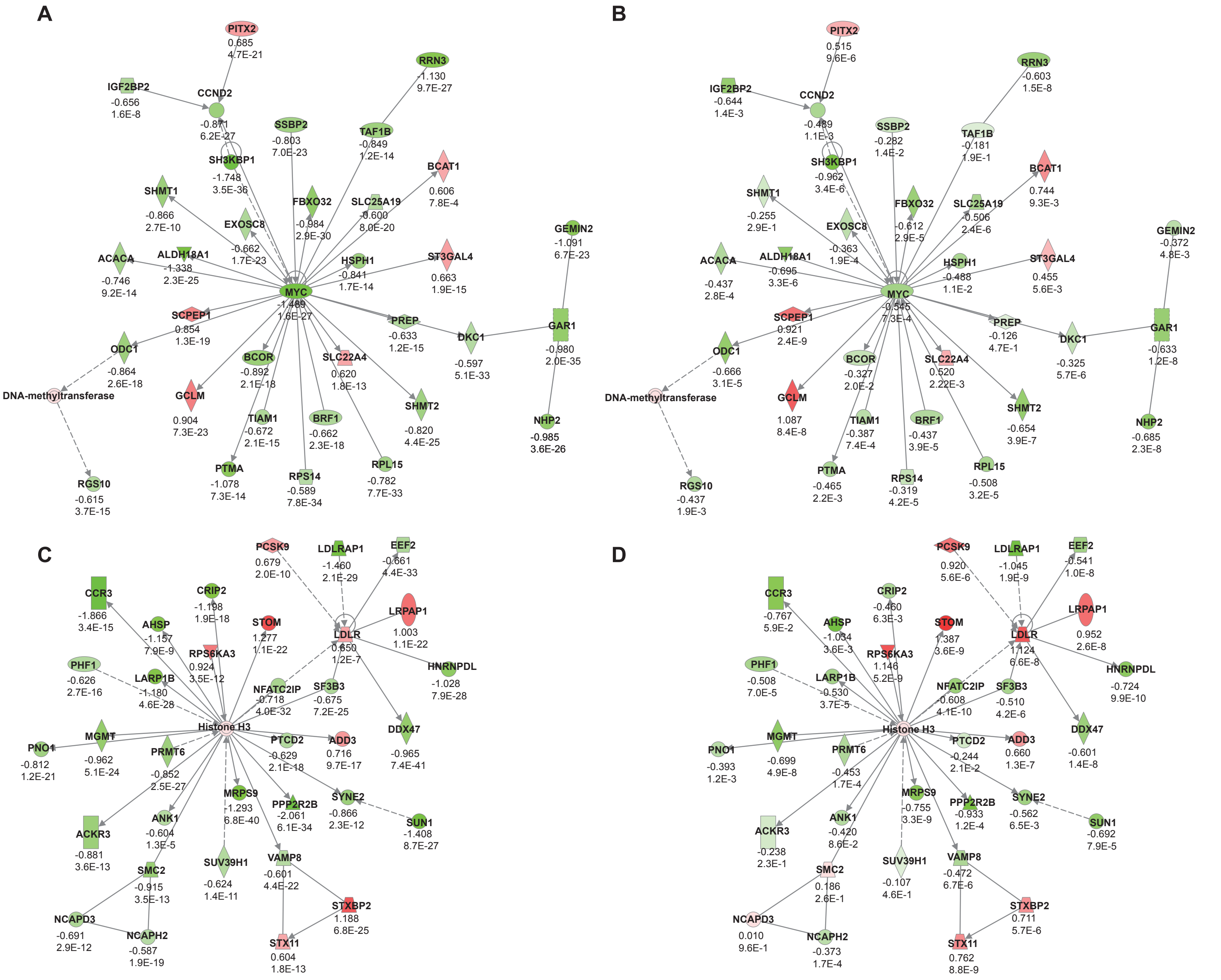

Figure S5. MYC and histone related networks identified among differentially expressed genes between patient SRS groups. In each network log fold change and FDR are shown below molecular symbols. (**A**) Gene network identified on network analysis of genes differentially expressed between SRS groups in the discovery cohort included 34 genes with MYC as the node (second most significant network identified, P 1x10^{-27}). MYC, a transcription factor involved in cell proliferation, apoptosis and survival, was significantly down-regulated in SRS1 patients, as was the anti-apoptotic gene BCL2, overexpression of which improves survival in sepsis[18]. Expression of the serine/threonine kinase RIP3 (RIPK3) which plays an essential role in necroptosis[19] and its substrate MLKL were increased in SRS1 patients, while BIRC3 encoding cIAP2, a critical inhibitor of necroptosis conferring protection during influenza virus infections[20], was down-regulated. (**B**) Gene expression data for differentially expressed genes in the validation cohort overlaid on the same MYC gene network. (**C**) Gene network identified on network analysis of differentially expressed genes between SRS groups in the discovery cohort included 34 genes with histone H3 gene complex as the node (P 1x10^{-27}). The majority of assayed histone genes were up-regulated in SRS1 patients (all 7 genes with >1.5 FC up-regulated). Extracellular histones have potent toxic effects, notably in the context of neutrophil extracellular traps (NETs) resulting in NETosis, which in the context of sepsis can promote endothelial dysfunction, hypoxia and death[21]. (**D**) Gene expression data for differentially expressed genes in the validation cohort overlaid on the same histone gene network.

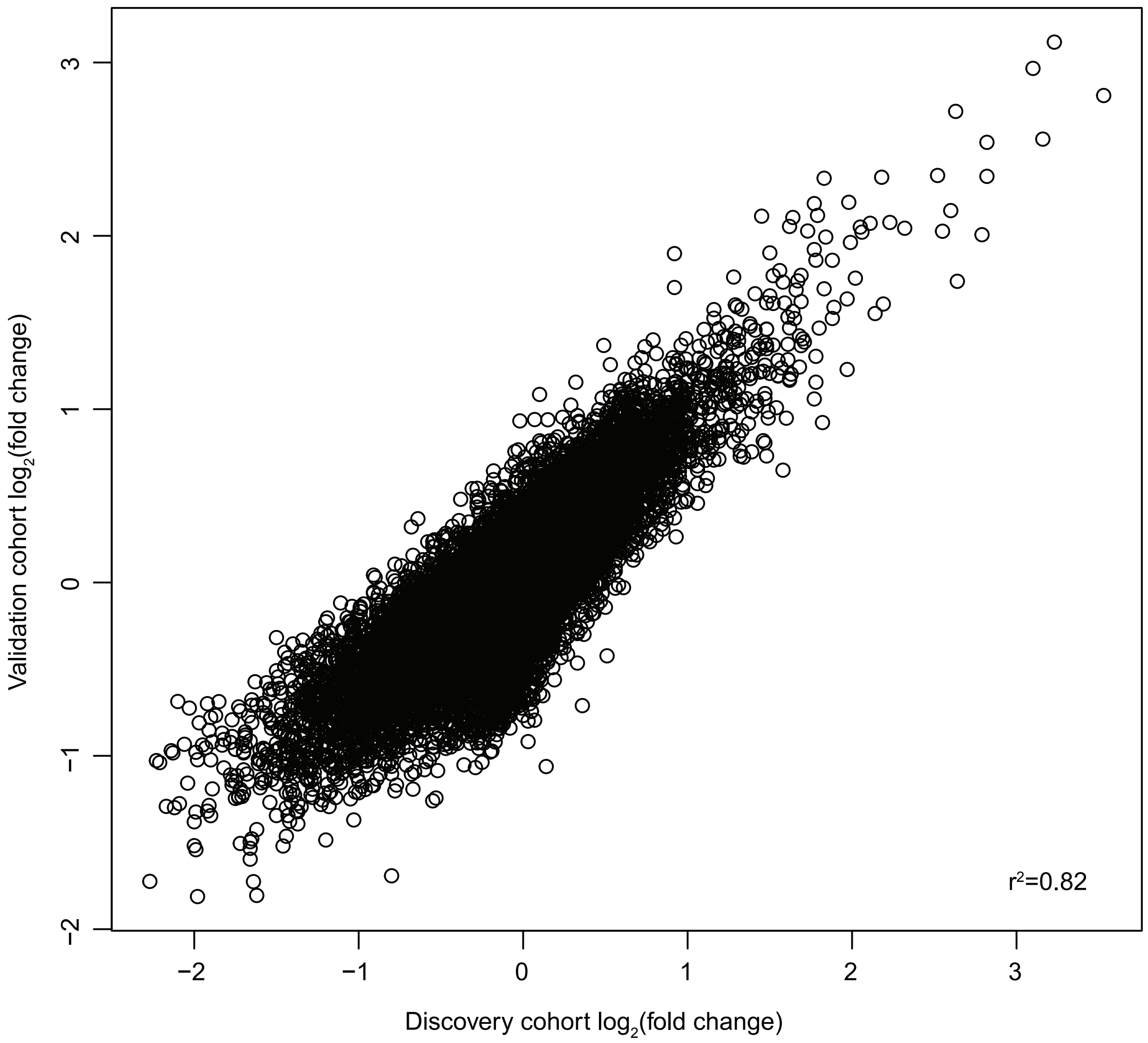

Figure S6. Correlation of differential gene expression between SRS groups in derivation and validation cohorts. The log fold change for differential gene expression of all measured genes between SRS groups 1 and 2 in the validation cohort is plotted against log fold change for the same genes between SRS groups 1 and 2 in the discovery cohort. The log fold changes are highly correlated (r^2 0.82 P $<2.2x10^{-16}$).

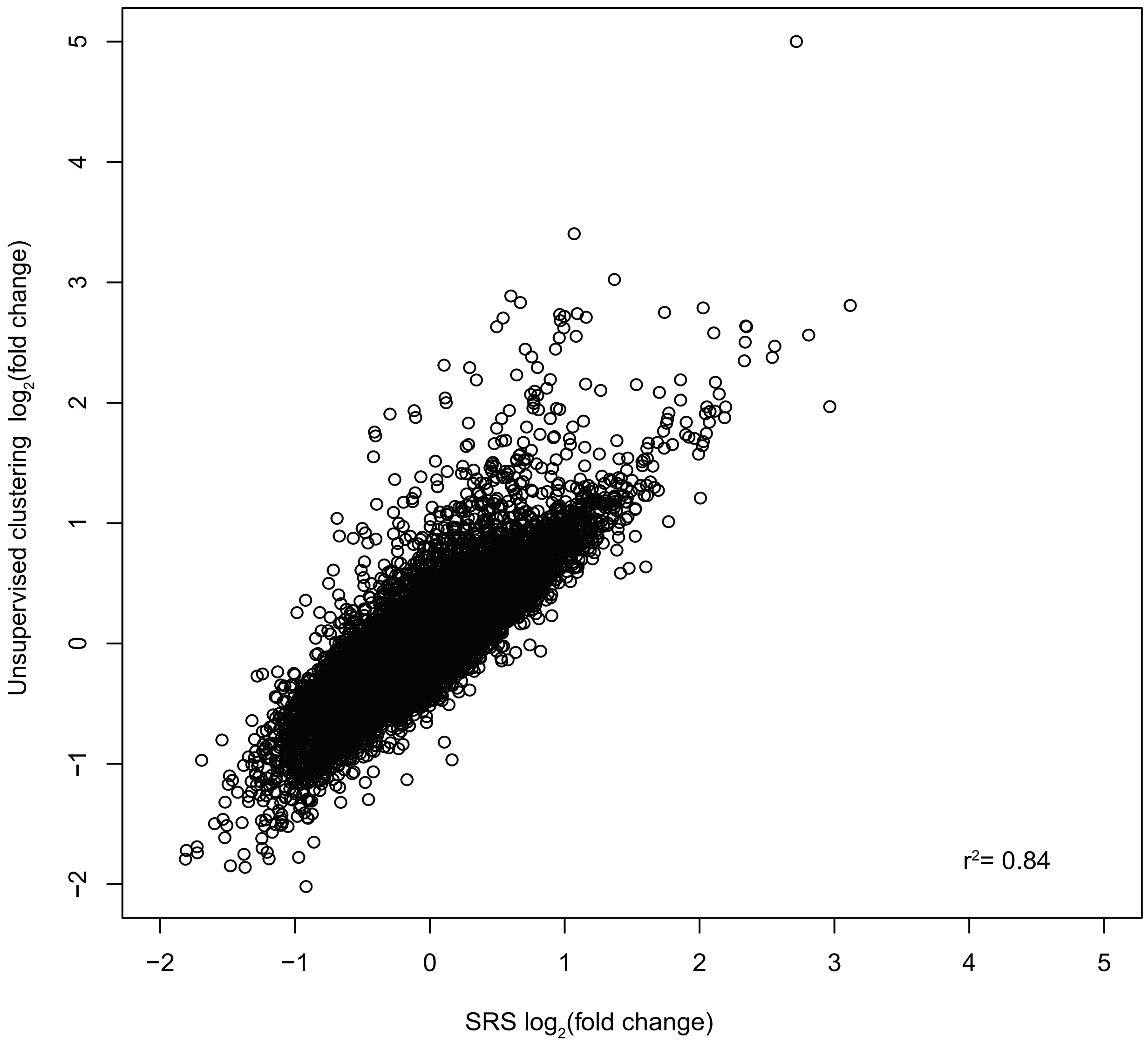

Figure S7. Correlation of differential gene expression between groups in the validation cohort determined using the SRS predictive gene set or an unsupervised clustering approach. The log fold change for differential gene expression of all measured genes between groups determined by unsupervised clustering in the validation cohort is plotted against log fold change for the same genes between SRS groups 1 and 2 defined by our predictive gene set in the same cohort. The log fold changes are highly correlated (r^2 0.84 P $<2.2\text{x}10^{-16}$).

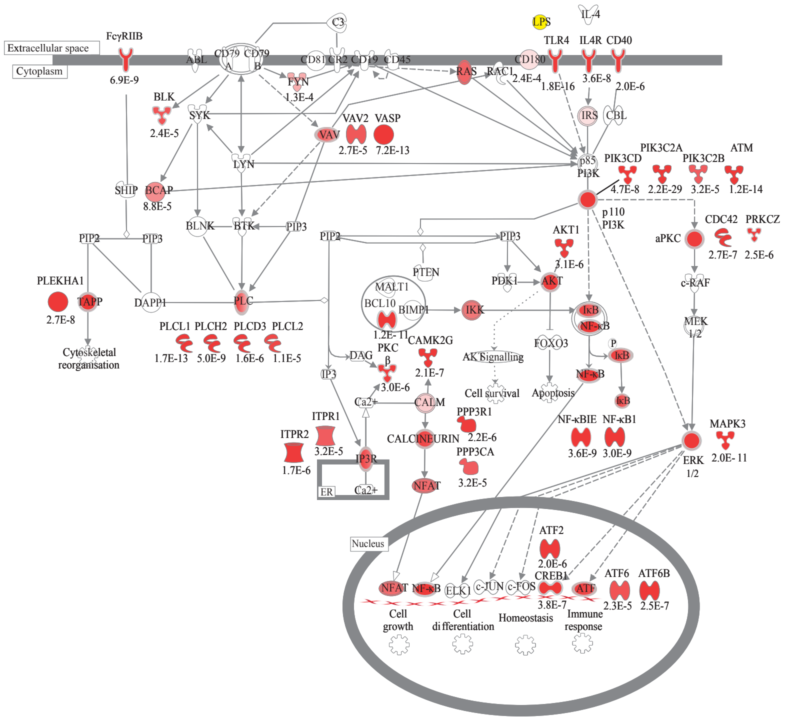

Figure S8. PI3K signalling canonical pathway enrichment for sepsis cis-eQTL. Genes showing eQTL shaded red with P values for cis-eQTL shown below. IPA canonical pathway analysis showed significant enrichment (P 3.77x10^{-4}) for PI3K signalling involving 28 molecules.

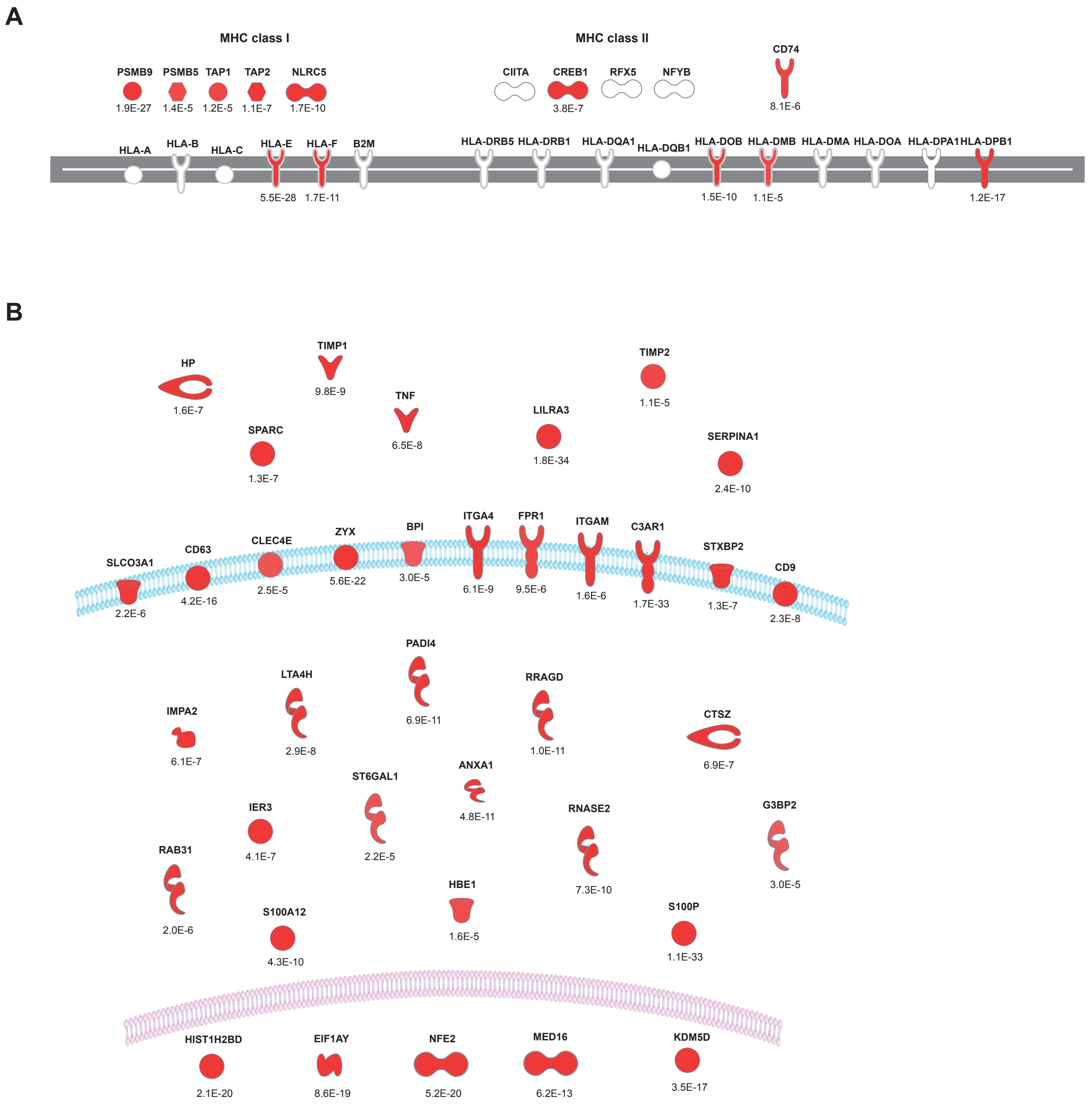

Figure S9. Pathway enrichment for sepsis cis-eQTL (FDR <0.01). Genes showing eQTL shaded red with P values for cis-eQTL shown below. **(A)** MHC genes and antigen presentation. IPA canonical pathway analysis showed significant enrichment (P 5.3×10^{-4}) for antigen presentation involving 12 molecules. Figure shows classical HLA molecules and associated proteins. There is evidence of eQTL involving antigen loading/processing genes including TAP, invariant chain (*CD74*) as well as eQTL involving classical class I and II molecules, and regulators such as *NLRC5*. **(B)** Viral respiratory infection. Analysis of IPA disease pathways showed most significant enrichment for viral respiratory infection (P 3.4×10^{-8}) involving 37 molecules.

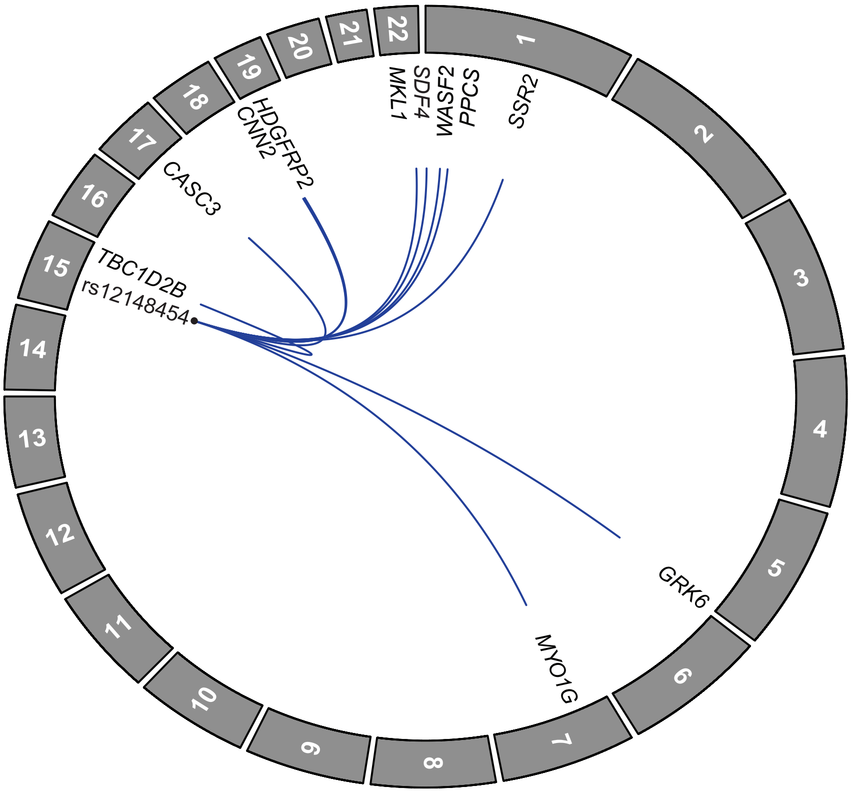

Figure S10. Trans-eQTL gene hub involving rs12148454. Circos plot showing trans-eQTL for rs12148454. Blue lines show trans associations (FDR < 0.05) with gene names indicated. This SNP shows a cis-eQTL (P 2.9×10^{-7}) for *VPS18* encoding vacuole protein sorting 18, a subunit of the homotypic fusion and vacuole protein sorting complex (HOPS) involved in endocytic and autophagocytic pathways. *VPS18* has been shown to be critical to interactions between signalling and membrane trafficking[22]. Related trans associated genes include *WASF2* encoding a WAS protein family member involved in transduction of signals relating to cell shape, motility and function; *SSR2*, encoding signal sequence receptor 2; and *MYO1G*, encoding plasma membrane myosin IG which is abundant in lymphocytes and involved in Fc-gamma receptor mediated phagocytosis.

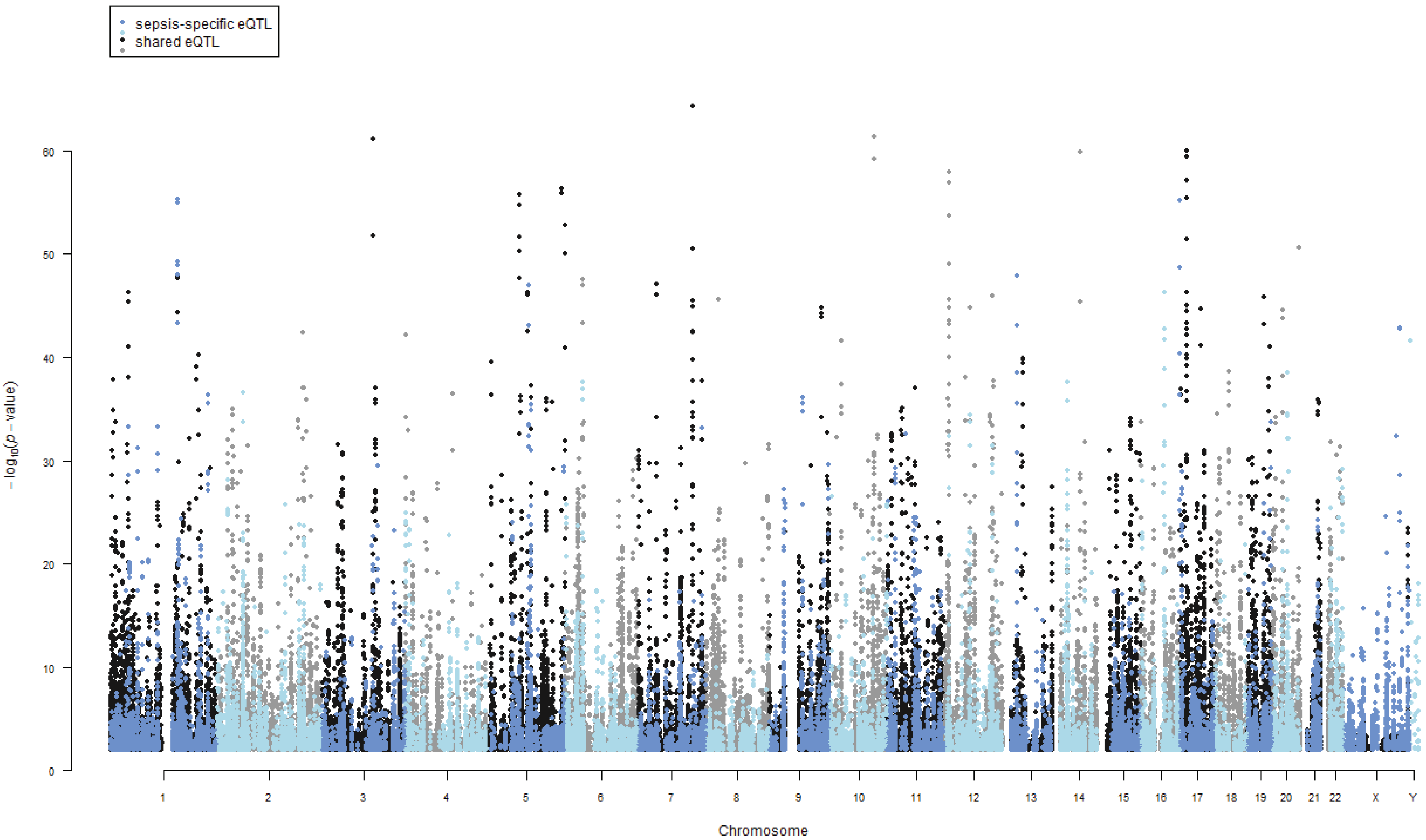

Figure S11. Sepsis-specific cis-eQTL. Manhattan plot showing for sepsis cis-eQTL those that are shared and those that are specific compared to a whole blood eQTL meta-analysis by Westra et al[15]. Blue and light blue show eSNPs only seen in sepsis; black and grey show eSNPs identified in sepsis patients that were also present in whole blood eQTL.

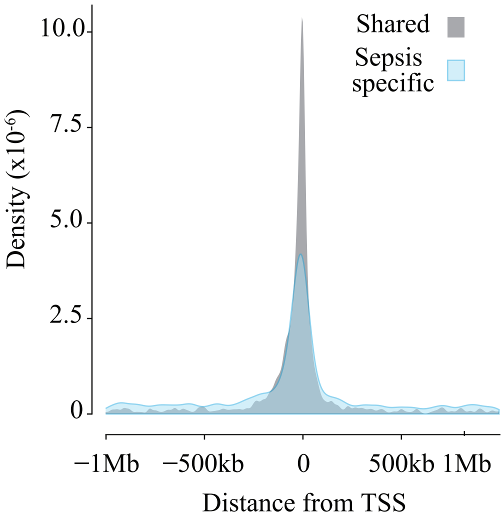

Figure S12. eQTL shared or specific to sepsis and distance from the transcription start site (TSS).

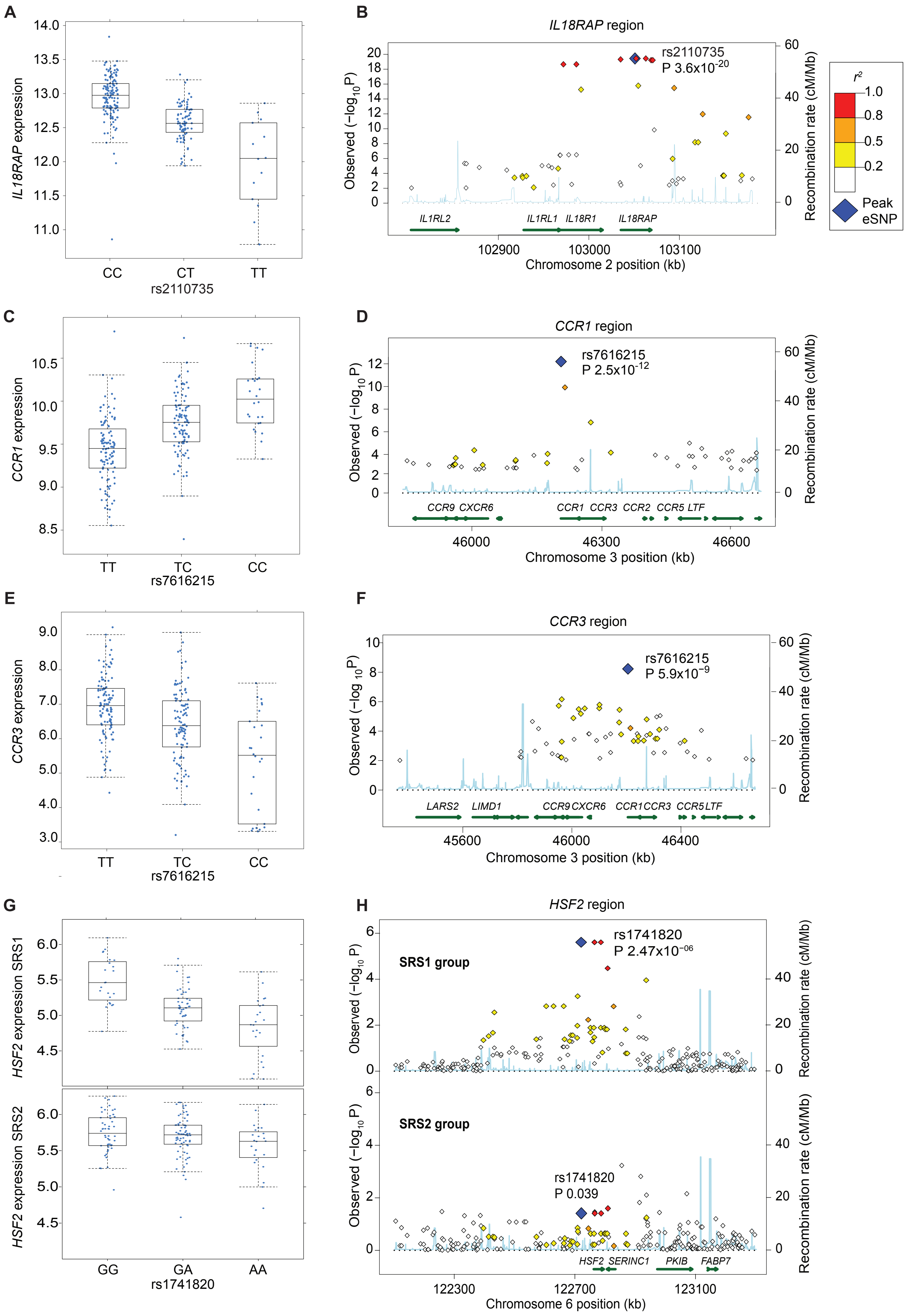
A
IL18RAP expression
CC
CT
TT
rs2110735
B
IL18RAP region
rs2110735
P 3.6x10^-20
Observed (−log10P)
Recombination rate (cM/Mb)
IL1RL2
IL1RL1
IL18R1
IL18RAP
Chromosome 2 position (kb)
r²
Peak eSNP
C
CCR1 expression
TT
TC
CC
rs7616215
D
CCR1 region
rs7616215
P 2.5x10^-12
CCR9 CXCR6
CCR1 CCR3 CCR2 CCR5 LTF
Chromosome 3 position (kb)
E
CCR3 expression
rs7616215
F
CCR3 region
rs7616215
P 5.9x10^-9
LARS2
LIMD1
CCR9 CXCR6
CCR1 CCR3
CCR5 LTF
Chromosome 3 position (kb)
G
HSF2 expression SRS1
HSF2 expression SRS2
GG
GA
AA
rs1741820
H
HSF2 region
SRS1 group
rs1741820
P 2.47x10^-06
SRS2 group
rs1741820
P 0.039
HSF2 SERINC1
PKIB
FABP7
Chromosome 6 position (kb)

Figure S13

Figure S13. Sepsis cis-eQTL involving genes differentially expressed between SRS groups and SRS-specific eQTL. Cis-eQTL illustrated by boxplots showing expression by genotype **(A,C,E,G)** and local regional association plots **(B,D,F,H)**. **(A-B)** *IL18RAP* shows evidence of a cis-eQTL involving rs2110735 (P $3.6x10^{-20}$ FDR $1.7x10^{-16}$) and is up-regulated in SRS1 patients (FC 1.9, FDR $3.2x10^{-22}$). **(C-F)** rs7616215 is the lead eSNP for cis-eQTL involving chemokine receptor genes *CCR1* (P $2.5x10^{-12}$ FDR $3.7x10^{-9}$) and *CCR3* (P $5.9x10^{-9}$ FDR $4.8x10^{-6}$). *CCR1* is significantly up-regulated in SRS1 patients (FC 1.8 FDR $3.1x10^{-14}$) while *CCR3* is significantly down-regulated (FC 0.3 FDR $3.4x10^{-15}$). **(G-H)** *HSF2* shows evidence of an eQTL specific to SRS1 involving rs1741820 (SRS1 P $2.47x10^{-6}$ FDR 0.0040 SRS2 P 0.039 FDR 0.74)

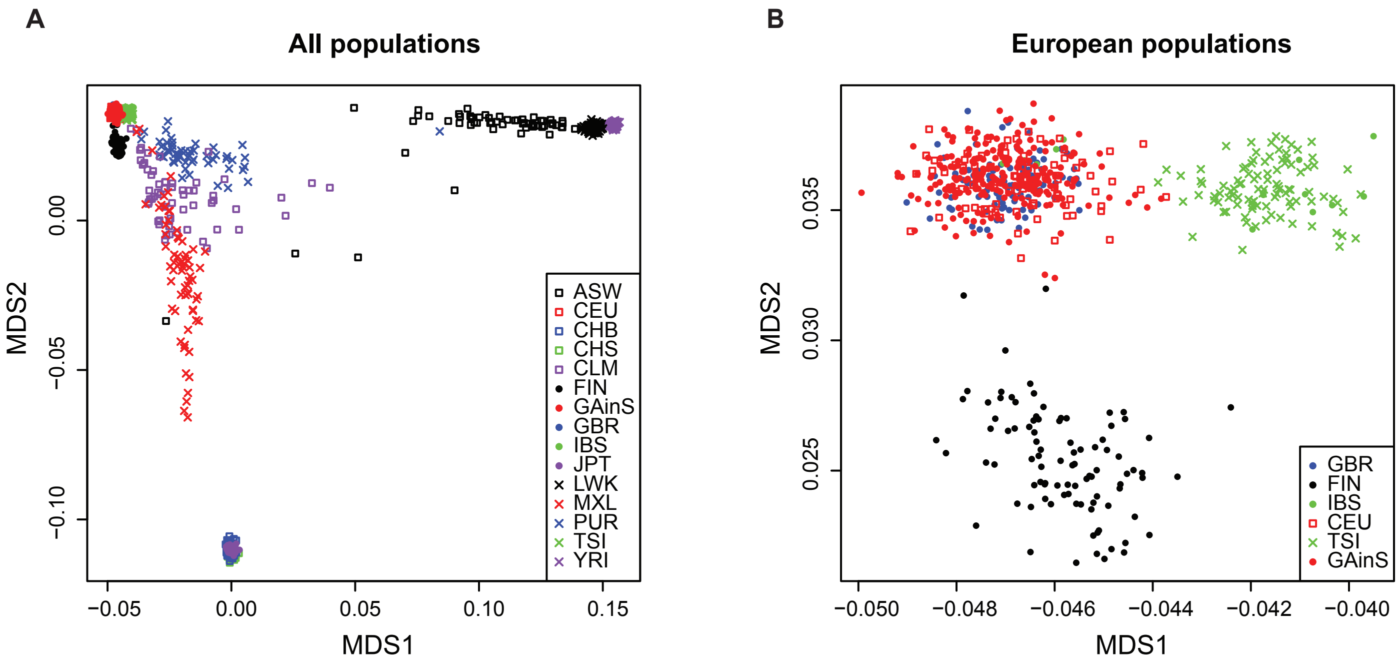

Figure S14. MDS plots comparing the genetic ancestry of sepsis (GAinS) patients to 1000 Genomes Project populations. The sepsis (GAinS) cohort is restricted to the 240 patients used for the eQTL analysis. **(A)** All populations. **(B)** European populations.

Table S1. Participating hospitals involved in patient recruitment and GAinS Investigators

Site	Principal Investigator	Research Nurses/Fellows	Other investigators
St Bartholomew's/Royal London Hospitals	Professor Charles Hinds	Eleanor McLees	Dr Chris Garrard, Dr D. Watson
John Radcliffe Hospital, Oxford	Dr Stuart McKechnie	Paula Hutton, Penny Parsons, Alex Smith	Dr Julian Millo, Dr Duncan Young
Royal Victoria Infirmary, Newcastle	Dr Simon Baudouin	Charley Higham, Helen Walsh	
Queen Elizabeth Hospital, Birmingham	Professor Julian Bion	Joanne Millar, Elsa Jane Perry	
Southend Hospital	Dr David Higgins	Sarah Andrews	
Leicester Royal Infirmary	Dr Jonathan Thompson	Sarah Bowrey, Sandra Kazembe	
Broomfield Hospital, Chelmsford	Dr D Arawwawala	Karen Swan, Sarah Williams, Susan Smolen	Dr Alasdair Short
Royal Berkshire Hospital, Reading	Dr Atul Kapila	Nicola Jacques, Jane Atkinson, Abby Brown	
University College London Hospital (UCLH), London	Dr Geoffrey Bellingan	Jung Hyun Ryu, Georgia Bercades	Dr Richard Marshall, Dr Hugh Montgomery
Norfolk & Norwich University Hospital, Norwich	Dr Simon Fletcher	Melissa Rosbergen, Georgina Glister	
Wythenshawe Hospital, Manchester	Dr Andrew Bentley	Katie Mccalman	
The James Cook University Hospital, Middlesbrough	Dr Stephen Bonner	Keith Hugill, Victoria Goodridge	
Aberdeen Royal Infirmary	Professor Nigel Webster	Jane Taylor, Sally Hall, Jenni Addison	Dr Helen Galley
Royal Hallamshire and Northern General Hospitals, Sheffield	Dr Gary Mills	John Humphreys, Kelsey Armitage	
St Mary's Hospital, London	Dr Martin Stotz	Adaeze Ochelli-Okpue	
Royal Sussex County Hospital, Brighton	Dr Stephen Drage	Laura Ortiz-Ruiz De Gordoa	
Charing Cross Hospital, London	Dr Tony Gordon	Emily Errington, Maie Templeton	
The Whittington Hospital, London	Dr Martin Kuper	Sheik Pahary	
Huddersfield Royal Infirmary	Dr Peter Hall	Jackie Hewlett	
Coventry and Warwickshire University Hospital, Coventry	Dr Pyda Venkatesh	Geraldine Ward, Marie McCauley	
North Middlesex Hospital, London	Dr Jeronimo Moreno Cuesta		
Hammersmith Hospital, London	Dr Stephen Brett	David Kitson	
Freeman Hospital, Newcastle	Dr Simon Baudouin	Charley Higham	
Royal Preston hospital, Preston	Dr Shond Laha	Jacqueline Baldwin, Angela Walsh	
Blackpool Victoria Hospital	Dr Achyut Guleri	Natalia Waddington	
Royal Blackburn Hospital	Dr Anton Krige	Martin Bland, Lynne Bullock, Donna Harrison	
Kettering General Hospital	Dr Jasmeet Soar	Parizade Raymode	
Southmead Hospital, Bristol	Dr Jasmeet Soar	Sally Grier	
Frenchay Hospital, Bristol	Dr Jasmeet Soar	Sally Grier	
